# Hyperglycemia Increases Severity of Staphylococcus aureus Osteomyelitis and Influences Bacterial Genes Required for Survival in Bone

**DOI:** 10.1128/iai.00529-22

**Published:** 2023-03-06

**Authors:** Casey E. Butrico, Nathan Klopfenstein, Erin R. Green, Joshua R. Johnson, Sun H. Peck, Carolyn B. Ibberson, C. Henrique Serezani, James E. Cassat

**Affiliations:** a Department of Pathology, Microbiology, and Immunology, Vanderbilt University Medical Center, Nashville, Tennessee, USA; b Vanderbilt Center for Bone Biology, Vanderbilt University Medical Center, Nashville, Tennessee, USA; c Division of Clinical Pharmacology, Department of Medicine, Vanderbilt University Medical Center, Nashville, Tennessee, USA; d Nashville VA Medical Center, Department of Veterans Affairs, Nashville, Tennessee, USA; e Department of Biomedical Engineering, Vanderbilt University, Nashville, Tennessee, USA; f Department of Microbiology and Plant Biology, The University of Oklahoma, Norman, Oklahoma, USA; g Department of Medicine, Division of Infectious Diseases, Vanderbilt University Medical Center, Nashville, Tennessee, USA; h Vanderbilt Institute for Infection, Immunology, and Inflammation (VI4), Vanderbilt University Medical Center, Nashville, Tennessee, USA; i Department of Pediatrics, Division of Pediatric Infectious Diseases, Vanderbilt University Medical Center, Nashville, Tennessee, USA; New York University Grossman School of Medicine

**Keywords:** osteomyelitis, hyperglycemia, superoxide dismutase, transposon sequencing, *Staphylococcus aureus*

## Abstract

Hyperglycemia, or elevated blood glucose, renders individuals more prone to developing severe Staphylococcus aureus infections. S. aureus is the most common etiological agent of musculoskeletal infection, which is a common manifestation of disease in hyperglycemic patients. However, the mechanisms by which S. aureus causes severe musculoskeletal infection during hyperglycemia are incompletely characterized. To examine the influence of hyperglycemia on S. aureus virulence during invasive infection, we used a murine model of osteomyelitis and induced hyperglycemia with streptozotocin. We discovered that hyperglycemic mice exhibited increased bacterial burdens in bone and enhanced dissemination compared to control mice. Furthermore, infected hyperglycemic mice sustained increased bone destruction relative to euglycemic controls, suggesting that hyperglycemia exacerbates infection-associated bone loss. To identify genes contributing to S. aureus pathogenesis during osteomyelitis in hyperglycemic animals relative to euglycemic controls, we used transposon sequencing (TnSeq). We identified 71 genes uniquely essential for S. aureus survival in osteomyelitis in hyperglycemic mice and another 61 mutants with compromised fitness. Among the genes essential for S. aureus survival in hyperglycemic mice was the gene encoding superoxide dismutase A (*sodA*), one of two S. aureus superoxide dismutases involved in detoxifying reactive oxygen species (ROS). We determined that a *sodA* mutant exhibits attenuated survival *in vitro* in high glucose and *in vivo* during osteomyelitis in hyperglycemic mice. SodA therefore plays an important role during growth in high glucose and promotes S. aureus survival in bone. Collectively, these studies demonstrate that hyperglycemia increases the severity of osteomyelitis and identify genes contributing to S. aureus survival during hyperglycemic infection.

## INTRODUCTION

Hyperglycemia, or elevated blood glucose, can result from chronic metabolic conditions as well as acute stress and reflects the inability of the body to produce or effectively use insulin. Hyperglycemia leads to a variety of physiological and immune regulatory alterations (1–6). Dysregulated cytokine response, altered chemotaxis, and decreased function of innate immune cells contribute to a hyperinflammatory environment and influence the pathophysiology of bacterial infections in hyperglycemic hosts (1, 4, 7). Hyperglycemia also increases oxidative stress in tissues via production of reactive oxygen species (ROS), which are generated as a consequence of electron transport chain dysfunction during mitochondrial respiration and glucose metabolism (8). The combination of ineffective immune responses and hyperinflammation leads to a greater incidence of infection in individuals with hyperglycemia (9). Staphylococcus aureus is a particularly common etiologic agent of severe infections in patients with hyperglycemia. Infection of bone, or osteomyelitis, is one of the most frequent manifestations of invasive staphylococcal infection in these patients (10). S. aureus osteomyelitis is particularly difficult to treat due to widespread antibiotic resistance, antibiotic tolerance, and the induction of bone destruction that can limit antibiotic delivery to the infectious focus (11, 12). Additionally, individuals with chronic hyperglycemia have greater bone porosity, higher rates of osteoporosis, and enhanced risk of bone fractures, further complicating treatment (13, 14). However, how hyperglycemia alters osteomyelitis pathogenesis or bacterial adaptation to the host microenvironment during osteomyelitis is not fully understood.

S. aureus has a remarkable ability to infect a variety of tissues, which can be attributed to its metabolic flexibility and the capacity to produce an arsenal of virulence factors (15–18). Multiple transcriptional regulators allow S. aureus to modulate its virulence in response to environmental cues, such as glucose abundance (17). However, the virulence and metabolic mechanisms by which S. aureus adapts to the altered host environment during osteomyelitis in hyperglycemic mice are not well understood. We hypothesized that the alterations in glucose availability and changes in host physiology during hyperglycemia require S. aureus to use distinct genes to survive and induce severe disease in the context of osteomyelitis.

To investigate the requirements for S. aureus survival and virulence *in vivo* during hyperglycemia, we subjected mice to acute and chronic hyperglycemia and then induced osteomyelitis using a posttraumatic model. Hyperglycemic mice exhibited more severe infection as measured by bacterial CFU and bone destruction. We used transposon sequencing (TnSeq) to identify S. aureus genes required for infection in hyperglycemic osteomyelitis. Based on the TnSeq analysis, we further studied the influence of superoxide dismutase A (SodA) on disease pathogenesis due to the known role of SodA in detoxifying ROS (19). SodA was found to be important for S. aureus survival in hyperglycemic mice. To study the mechanistic basis of this finding, we analyzed the growth of a *sodA* mutant *in vitro* in high glucose and identified that the *sodA* mutant exhibited a survival defect in high glucose. Collectively, the findings of this study uncover mechanisms of increased virulence during S. aureus osteomyelitis in hyperglycemic mice.

## RESULTS

### Acute hyperglycemia increases S. aureus burdens during osteomyelitis.

**FIG 1:**
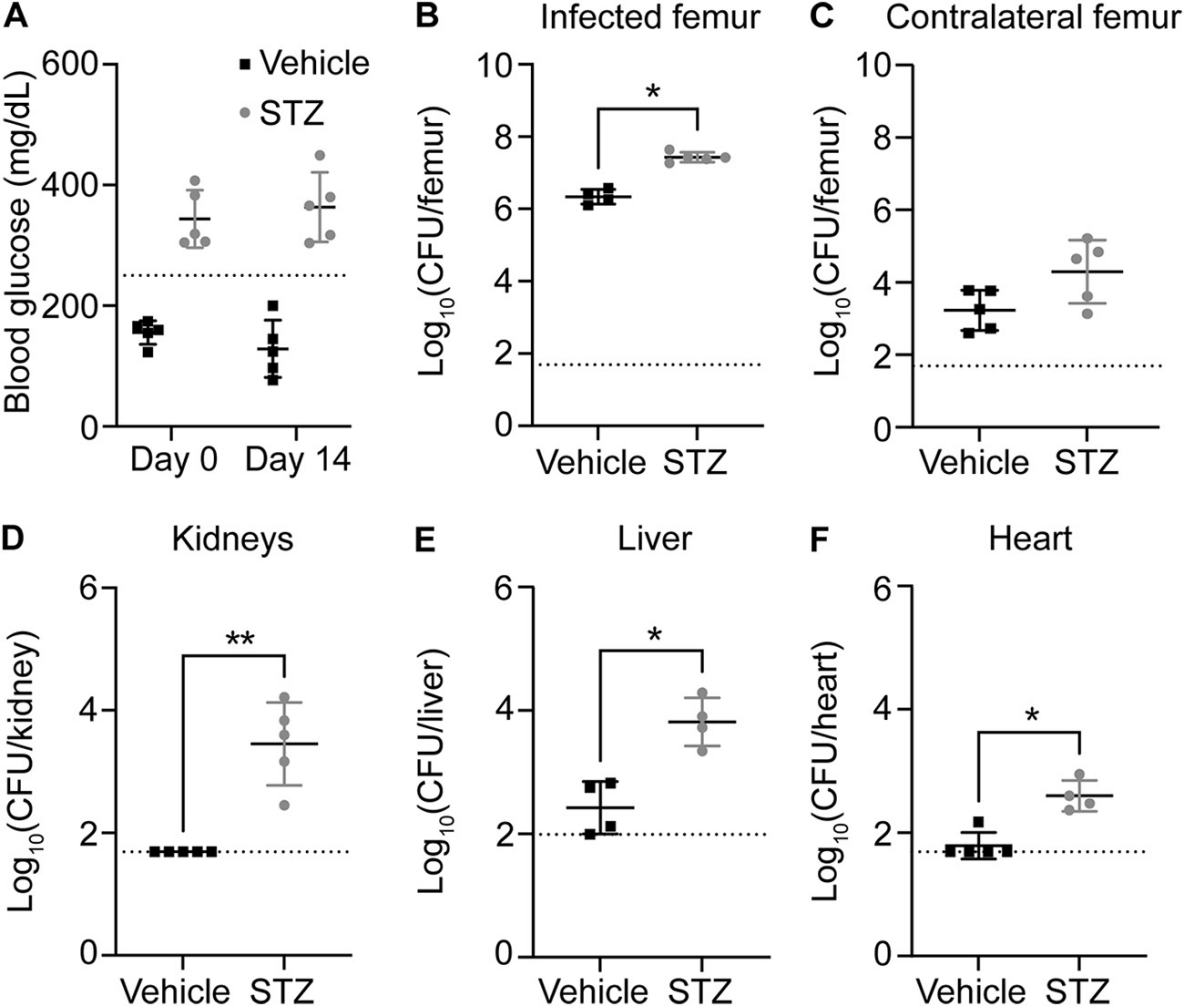
Acute hyperglycemia increases S. aureus burdens and dissemination during osteomyelitis. Eight-week-old male mice were treated with sodium citrate (vehicle) or streptozotocin (STZ) intraperitoneally for 5 days. Ten days after the final injection, mice were infected with 1 × 10^6^ CFU of WT S. aureus via intraosseous injection. (A) Blood glucose concentration was quantified from a tail vein bleed immediately prior to inoculation (day 0) and on the day of sacrifice (day 14). The dotted line indicates the hyperglycemia threshold of 250 mg/dL. (B to F) Mice were sacrificed at day 14 postinfection, and the bacterial burdens (CFU) were enumerated in infected femur (B), contralateral femur (C), kidneys (D), liver (*n* = 4 mice per group) (E), and heart (*n* = 4 mice in STZ-treated group) (F). One experiment was conducted with *n* = 5 mice per group unless otherwise noted. Dotted lines indicate limit of detection. Horizontal lines indicate means, and error bars represent SD. Significance was determined with the Mann-Whitney test (B to F). *, *P* < 0.05; **, *P* < 0.01.

To investigate the impact of hyperglycemia on S. aureus virulence during osteomyelitis, we first induced hyperglycemia in male C57BL/6J mice by treatment with streptozotocin (STZ) (7, 20). STZ induces hyperglycemia through cytotoxic effects on insulin-producing beta cells. We then subjected STZ- or vehicle-treated mice to osteomyelitis and determined S. aureus burdens following infection (18). Because the induction of hyperglycemia following STZ treatment is variable, we measured blood glucose levels in mice at the start (day 0) and end (day 14) of the infection (21, 22). In this experiment, all mice treated with STZ were hyperglycemic (defined as blood glucose levels of >250 mg/dL) at days 0 and 14 of infection (Fig. 1A). STZ-treated hyperglycemic mice and euglycemic vehicle-treated control mice were infected 10 days after the last STZ or vehicle treatment with 1 × 10^6^ CFU of S. aureus USA300 lineage strain AH1263 (wild type [WT]). To infect mice, we used a posttraumatic model of osteomyelitis, in which bacteria are inoculated directly into a cortical defect in the mid-femur (18, 23). Acute hyperglycemia resulted in a significant increase in S. aureus burdens in the infected femurs compared to those in euglycemic vehicle-treated animals at day 14 postinfection (Fig. 1B). To assess the extent to which hyperglycemia alters S. aureus dissemination to other tissues, we also collected the contralateral femur, kidneys, liver, and heart. While dissemination to the contralateral femur did not significantly change, dissemination to the kidneys, liver, and heart significantly increased in hyperglycemic animals (Fig. 1C to F). Similar trends were observed with a lower inoculum of S. aureus (see Fig. S1 in the supplemental material), although the only organs with significantly greater CFU in the infected hyperglycemic mice compared to euglycemic vehicle-treated were the infected femur and kidneys (Fig. S1B and C). These data suggest that acute hyperglycemia increases S. aureus survival in bone as well as dissemination to other organs during osteomyelitis.

To confirm that changes in S. aureus growth and dissemination result from acute hyperglycemia induced by STZ treatment and not from off-target effects of the drug, we assessed bacterial burdens in three additional groups of mice: euglycemic vehicle-treated mice, STZ-treated mice that became hyperglycemic, and STZ-treated mice that remained euglycemic (Fig. S2A). We inoculated mice with 1 × 10^6^ CFU of S. aureus and measured bacterial burdens in the femur 14 days postinfection. STZ-treated hyperglycemic mice had greater S. aureus burdens in infected femurs than both euglycemic vehicle-treated and euglycemic STZ-treated mice (Fig. S2B). These data indicate that changes in bacterial burden in STZ-treated mice result from hyperglycemia.

### Chronic hyperglycemia increases S. aureus burden during osteomyelitis.

**FIG 2:**
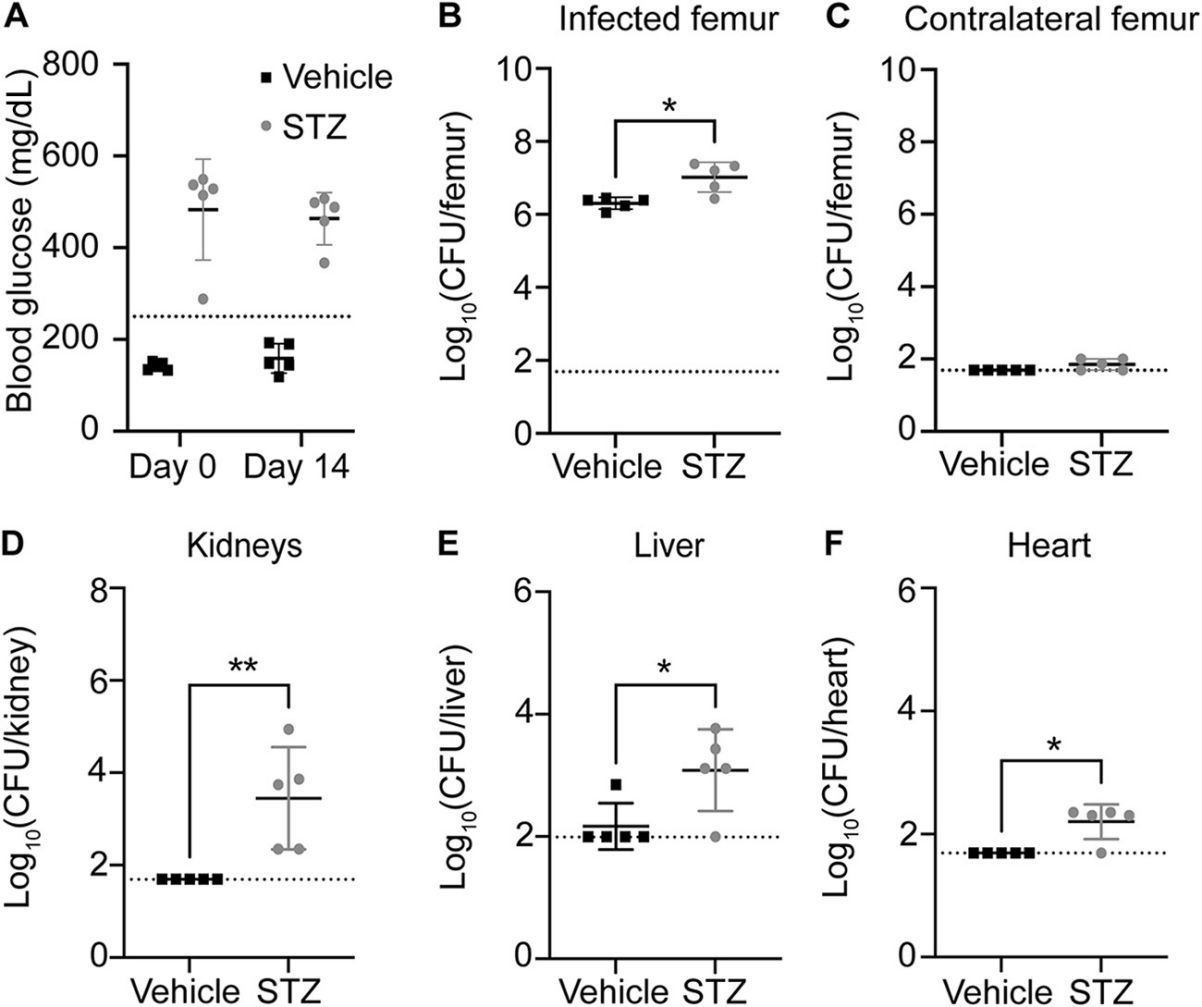
Chronic hyperglycemia increases S. aureus burdens and dissemination during osteomyelitis. Eight-week-old male mice were treated with sodium citrate (vehicle) or STZ intraperitoneally for 5 days. Thirty days after the final injection, mice were infected with 1 × 10^6^ CFU of WT S. aureus via intraosseous injection. (A) Blood glucose concentration was quantified from a tail vein bleed immediately prior to inoculation (day 0) and on the day of sacrifice (day 14). The dotted line indicates the hyperglycemia threshold of 250 mg/dL. (B to F) Mice were sacrificed at day 14 postinfection, and the bacterial burdens (CFU) were enumerated in infected femur (B), contralateral femur (C), kidneys (D), liver (E), and heart (F). One experiment was conducted with *n* = 5 mice per group. Dotted lines indicate limit of detection. Horizontal lines indicate means, and error bars represent SD. Significance was determined with the Mann-Whitney test (B to F). *, *P* < 0.05; **, *P* < 0.01.

Prior studies modeling S. aureus infection during chronic hyperglycemia have inoculated mice at 30 days after STZ treatment (1, 24). To determine if a more chronic state of hyperglycemia alters the pathogenesis of staphylococcal osteomyelitis, we treated mice with STZ for 5 days and then initiated osteomyelitis 30 days after the final STZ injection. Hyperglycemia was confirmed at the start (day 0) and end (day 14) of the infection (Fig. 2A). As observed with the acute model of hyperglycemia, bacterial burdens were elevated in infected femurs from hyperglycemic mice compared to euglycemic vehicle-treated mice at day 14 postinfection (Fig. 2B). Dissemination to contralateral femurs did not increase in hyperglycemic infected mice (Fig. 2C). However, S. aureus burdens in the kidneys, liver, and heart of the hyperglycemic infected mice were increased compared to those in euglycemic mice at day 14 postinfection (Fig. 2D to F). Similar trends were observed using a 10-fold-lower inoculum (Fig. S3). Taken together, these data suggest that both acute and chronic hyperglycemia result in increased S. aureus bacterial burdens during osteomyelitis.

### Hyperglycemia increases bone loss during S. aureus osteomyelitis.

**FIG 3:**
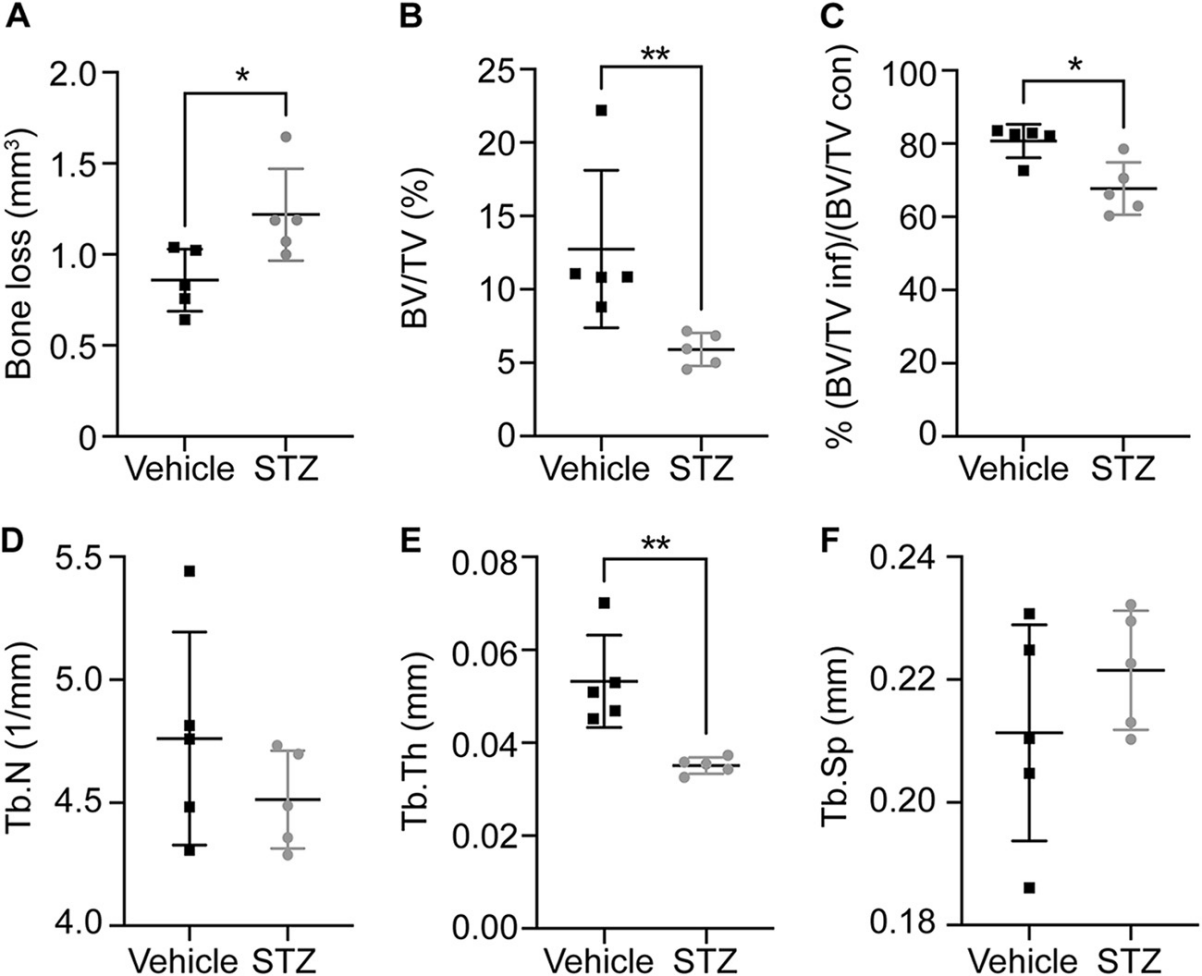
S. aureus incites greater bone destruction in acute hyperglycemic animals. Eight-week-old male mice were treated with sodium citrate (vehicle) or STZ intraperitoneally for 5 days. Ten days after the final injection, mice were infected with 1 × 10^5^ CFU of WT S. aureus via intraosseous injection. At 14 days postinfection, the infected femur and contralateral femur were isolated for microcomputed tomography. Cortical bone loss in infected femurs (A), trabecular bone volume divided by total volume (BV/TV) of infected femurs (B), and BV/TV of infected femurs relative to contralateral femurs (C) were quantified. Trabecular number (Tb.N) (D), trabecular thickness (Tb.Th) (E), and trabecular spacing (Tb.Sp) (F) were quantified in infected femurs. One experiment was conducted with *n* = 5 mice per group. Horizontal lines indicate means, and error bars represent SD. Significance was determined with the Mann-Whitney test. *, *P* < 0.05; **, *P* < 0.01.

To further characterize the pathogenesis of osteomyelitis during hyperglycemia, we measured bone loss following S. aureus infection. Significant bone damage and pathological remodeling occur during S. aureus osteomyelitis in euglycemic animals (18, 23, 25). Due to the increased S. aureus burdens observed in hyperglycemic mice compared to euglycemic mice, we hypothesized that bone destruction would increase in the setting of hyperglycemia. STZ-treated hyperglycemic mice and vehicle-treated euglycemic mice were inoculated with 1 × 10^5^ CFU of S. aureus due to the increased infection severity with a 1 × 10^6^ CFU inoculum. We used microcomputed tomography (μCT) to quantify changes to bone structure in the S. aureus-infected femurs at 14 days postinfection. The infected femurs from hyperglycemic mice had greater cortical bone loss relative to the infected femurs from euglycemic vehicle-treated mice during acute hyperglycemia (Fig. 3A). Similar trends in cortical bone loss were observed in infected mice with chronic hyperglycemia (Fig. S4A). To further characterize the impact of acute and chronic hyperglycemia on bone homeostasis, we also measured changes in the trabecular bone volume. At baseline, there was no difference in the trabecular bone volume relative to total volume (%BV/TV) between the hyperglycemic mice and the vehicle-treated euglycemic mice (Fig. S5). However, a decrease in %BV/TV was observed in the infected femurs from hyperglycemic mice compared to infected femurs from euglycemic vehicle-treated mice (Fig. 3B). Because chronic hyperglycemia has been shown to alter bone volume, we normalized the %BV/TV against the uninfected contralateral femur (14, 26). Importantly, the %BV/TV in the hyperglycemic infected femurs normalized against the %BV/TV of the contralateral femurs remained significantly lower in the hyperglycemic mice than in the euglycemic vehicle-treated mice, further suggesting that the trabecular bone loss in hyperglycemic mice was related to infection and not solely a function of baseline changes in bone volume (Fig. 3C). Trabecular bone thickness in infected hyperglycemic animals was lower than in the infected euglycemic vehicle-treated animals, with no differences observed in trabecular spacing or number (Fig. 3D to F). Similar trends in trabecular bone parameters were observed in the infected femurs of mice with chronic hyperglycemia (Fig. S4B to F). To further characterize tissue inflammation in hyperglycemic and euglycemic mice subjected to osteomyelitis, representative histological sections of S. aureus-infected femurs were stained with hematoxylin and eosin (H&E). Hyperglycemic infected femurs exhibited greater signs of inflammation than did the euglycemic vehicle-treated infected femurs (Fig. S6). Representative histological sections of infected femurs from chronic hyperglycemic mice exhibited pathology similar to that of acute hyperglycemic infected femurs (Fig. S7). Collectively, our data reveal that acute and chronic hyperglycemia contribute to greater pathological bone destruction during S. aureus osteomyelitis compared to euglycemic infection. Due to the similar findings between acute and chronic hyperglycemic mice, we performed further experiments using the acute model of hyperglycemia.

### Genes contributing to the fitness of S. aureus in hyperglycemic animals.

Hyperglycemia results in increased S. aureus burdens within infected femurs and greater dissemination to other organs in the context of osteomyelitis. To identify genes required for staphylococcal survival in hyperglycemic tissues, we performed TnSeq in hyperglycemic and euglycemic animals with a previously characterized USA300 LAC transposon insertion library (19). Groups of mice were treated with STZ or vehicle, and 10 days after the final treatment, mice were infected with 5 × 10^6^ CFU of the S. aureus transposon insertion library. An *in vitro* comparator consisted of growth in brain heart infusion (BHI) for 24 h to identify genes that are essential for bacterial growth under a nutrient-rich condition.

**FIG 4:**
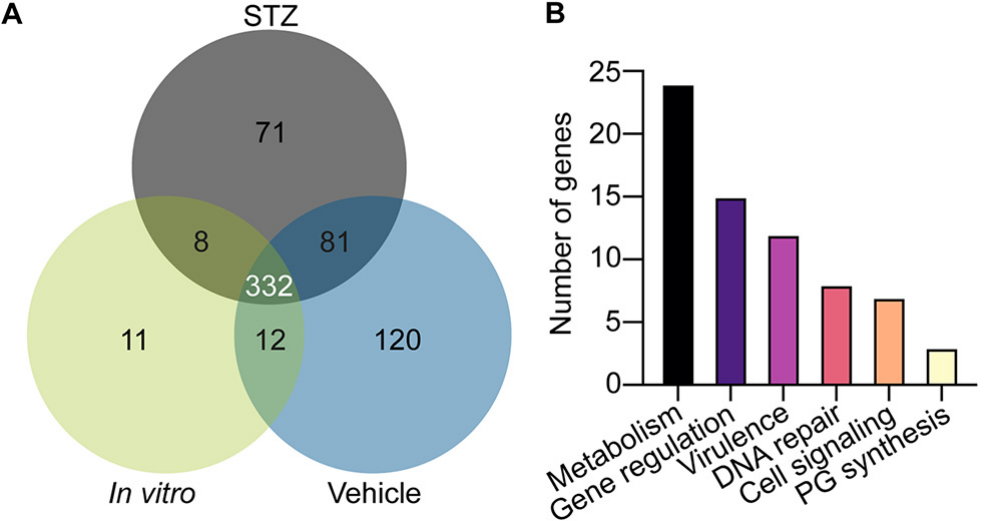
Transposon sequencing reveals genes essential for S. aureus survival during osteomyelitis in mice with hyperglycemia. Eight-week-old male mice were treated with sodium citrate (vehicle) or STZ intraperitoneally for 5 days. Ten days after the final injection, mice were infected with 5 × 10^6^ CFU of the S. aureus TnSeq library via intraosseous injection. At day 4 postinfection, bacteria were recovered and Illumina sequencing was used to identify the abundance of S. aureus mutants under each condition. (A) Based on Dval calculations, the number of essential genes (Dval < 0.01) *in vitro* in BHI, *in vivo* in STZ-treated hyperglycemic mice, and *in vivo* in euglycemic vehicle-treated mice were enumerated. (B) The annotated genes essential for S. aureus survival (Dval < 0.01) and the mutants with compromised fitness (Dval > 0.01 and Dval < 0.1) only under the condition of osteomyelitis during hyperglycemia were organized into categories of metabolism, gene regulation, virulence, DNA repair, cell signaling, and peptidoglycan (PG) synthesis. Two experiments were conducted with 6 mice per *in vivo* group per experiment, pooled in samples of 2 for *n* = 6 in sequencing analysis.

A TnSeq Dval score was assigned to each gene based on the number of reads within a given gene in a sample divided by the predicted number of reads for the gene considering its size and total sequencing reads for the given sample. TnSeq identified 71 S. aureus genes as essential (defined as Dval of <0.01) for survival during osteomyelitis in hyperglycemic mice but not essential for growth *in vitro* or in euglycemic mice (Fig. 4A and Table S1). We also identified 61 S. aureus transposon mutants with compromised fitness (Dval of >0.01 and <0.1) in hyperglycemic mice but not *in vitro* or in euglycemic mice (Table S2). Of these 132 genes, 45 encode hypothetical proteins; 28 of these genes are considered essential and 17 have compromised fitness. Of the remaining 87 genes, 61 have Kyoto Encyclopedia of Genes and Genomes (KEGG) identifiers. Of the 61 genes with KEGG identifiers, 24 are implicated in metabolic processes, including glycolysis, glutamine metabolism, histidine metabolism, and purine/pyrimidine biosynthesis. Genes related directly or indirectly to glucose metabolism that were identified as either essential or compromised during osteomyelitis include catabolite control protein A (*ccpA*, SAUSA300_1682), l-lactate dehydrogenase 1 (*ldh1*, SAUSA300_0235), dihydrolipoyl dehydrogenase (*lpdA*, SAUSA300_0996), and pyruvate ferredoxin oxidoreductase (SAUSA300_1182). Other metabolic genes identified as essential included carbamoyl phosphate synthase large subunit (*carB*, SAUSA300_1096), carbamoyl phosphate synthase small subunit (*carA*, SAUSA300_1095), and adenine phosphoribosyltransferase (*apt*, SAUSA300_1591).

In addition to metabolic pathways identified as important for S. aureus survival in osteomyelitis during hyperglycemia, the remaining genes identified by TnSeq are broadly involved in bacterial stress responses. These genes can be classified by roles in DNA repair, cell signaling, gene regulation, peptidoglycan synthesis, and virulence (Fig. 4B). One virulence gene identified as uniquely essential for S. aureus growth during osteomyelitis in hyperglycemic mice was *sodA* (SAUSA300_1513), which encodes superoxide dismutase A. SodA is responsible for detoxifying ROS via conversion of superoxide radicals to hydrogen peroxide (H_2_O_2_) (27). Due to the hyperinflammation during hyperglycemia and presence of greater ROS concentrations, we sought to understand how SodA facilitates S. aureus growth in the presence of elevated glucose (6).

### SodA facilitates S. aureus survival in high glucose *in vitro*.

**FIG 5:**
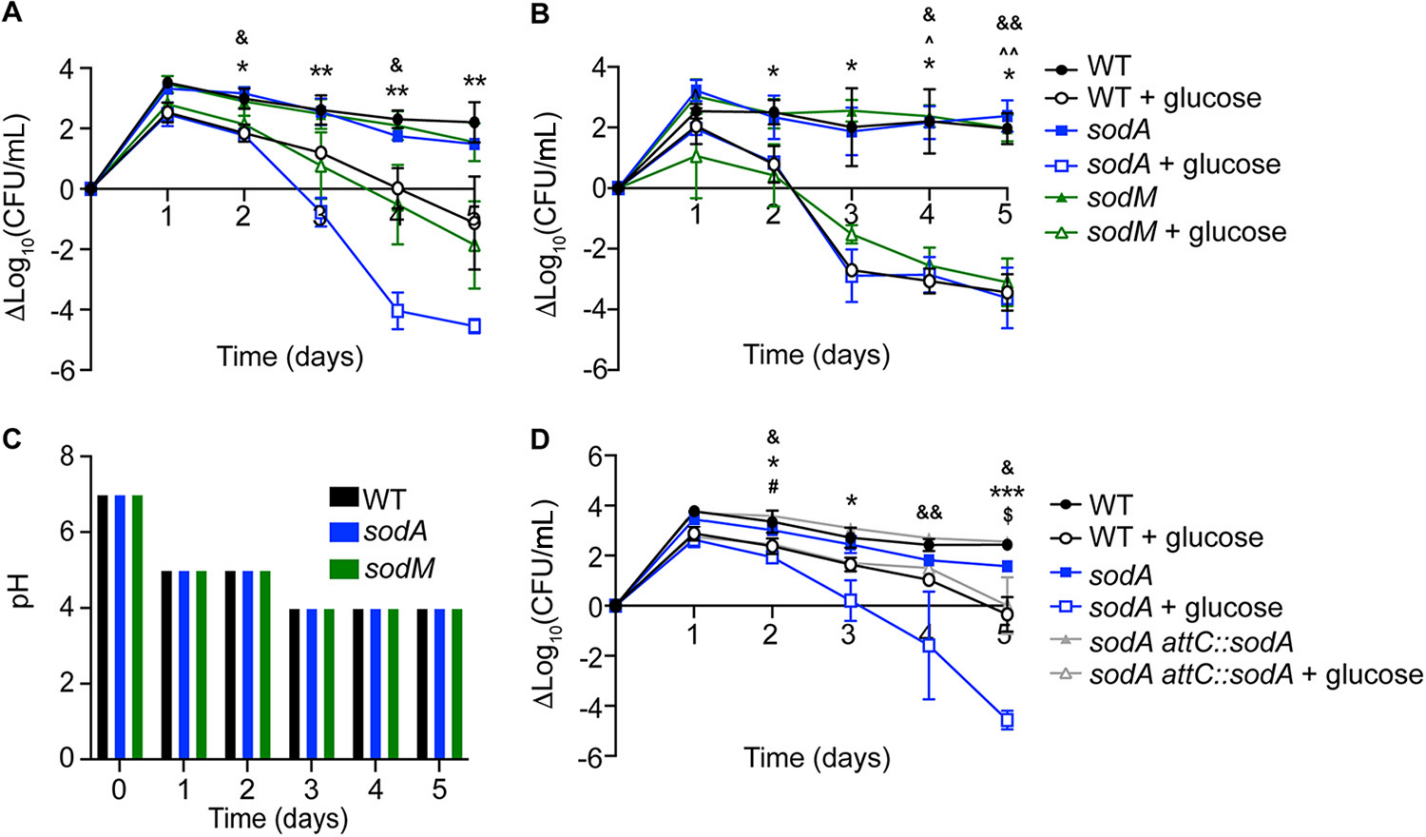
S. aureus *sodA* is required for survival during culture in high glucose. (A and B) WT, *sodA*::tet, and *sodM*::erm S. aureus strains were grown in 10 mL of TSB with and without 500 mg/dL of added glucose in flasks covered with foil (aerobic) (A) or capped (microaerobic) (B) with shaking at 37°C. CFU were quantified every 24 h over the course of 5 days and normalized to time zero. (C) pH was measured every 24 h over the course of 5 days in TSB with 500 mg/dL of glucose under aerobic and microaerobic conditions. (D) WT, *sodA*::tet, and *sodA*::tet *attC*::*sodA* S. aureus strains were grown in 10 mL of TSB and TSB with 500 mg/dL of glucose in flasks covered with foil (aerobic) (*n* = 2 technical replicates and *n* = 3 biological replicates). The line represents the mean, and error bars represent SD (A, B, and D). Significance was determined with two-way ANOVA and Dunnett’s multiple-comparison test. *, *P* < 0.05; **, *P* < 0.01; ***, *P* < 0.001 (*sodA*::tet TSB plus glucose). &, *P* < 0.05; &&, *P* < 0.01 (WT TSB plus glucose). ^, *P* < 0.05; ^^, *P* < 0.01 (*sodM*::erm TSB plus glucose). #, *P* < 0.05 (*sodA*::tet *attC*::*sodA* TSB plus glucose). $, *P* < 0.01 (*sodA*::tet TSB). All comparisons were made to the WT in TSB.

While host cells produce ROS during inflammation, S. aureus can also produce ROS intrinsically in response to high levels of glucose in the growth media (28). To combat ROS, the S. aureus genome includes genes for two superoxide dismutases (SODs), *sodA* and *sodM* (27, 29). The *sodA* gene was identified as essential for growth during osteomyelitis in hyperglycemic mice (Dval of 0.0003), while *sodM* was not essential for growth *in vitro* (Dval of 1.204), in euglycemic vehicle-treated osteomyelitis infection (Dval of 1.801), or in osteomyelitis in hyperglycemic mice (Dval of 0.6585). To evaluate the roles of each SOD in S. aureus survival in elevated glucose in the absence of other host stressors, we examined survival of WT, *sodA* mutant, and *sodM* mutant strains over 5 days in tryptic soy broth (TSB; 250 mg/dL of glucose) or in TSB with an additional 500 mg/dL of glucose by quantifying CFU every 24 h (28). While the WT and the *sodM* mutant survived in TSB and TSB with glucose to similar extents over the course of 5 days, the *sodA* mutant had a survival defect in TSB with glucose compared to WT by day 2 (Fig. 5A). Prior studies suggest that oxygen is critical for intrinsic ROS production as well as SOD activity, which led us to hypothesize that differences in survival between WT and the SOD mutants would be minimized under conditions of limited oxygen (27, 30). In keeping with this hypothesis, WT S. aureus grew similarly to the *sodA* mutant strain under microaerobic conditions (Fig. 5B). Although S. aureus cultures with high glucose became more acidic over time, changes in survival between S. aureus strains were not explained by differences in pH (Fig. 5C). However, the *sodA* mutant was unable to survive to the same extent as WT under low-pH conditions *in vitro*, which could partially explain the reduced fitness of this mutant in the presence of high glucose (Fig. S8). Complementation with *sodA* in *cis* rescued the survival the *sodA* mutant *in vitro* when grown aerobically in TSB with glucose (Fig. 5D). Consistent with prior studies, these findings support a role for SodA in detoxifying intrinsically generated superoxide (31, 32).

### SodA enhances S. aureus survival during osteomyelitis in hyperglycemic mice.

**FIG 6:**
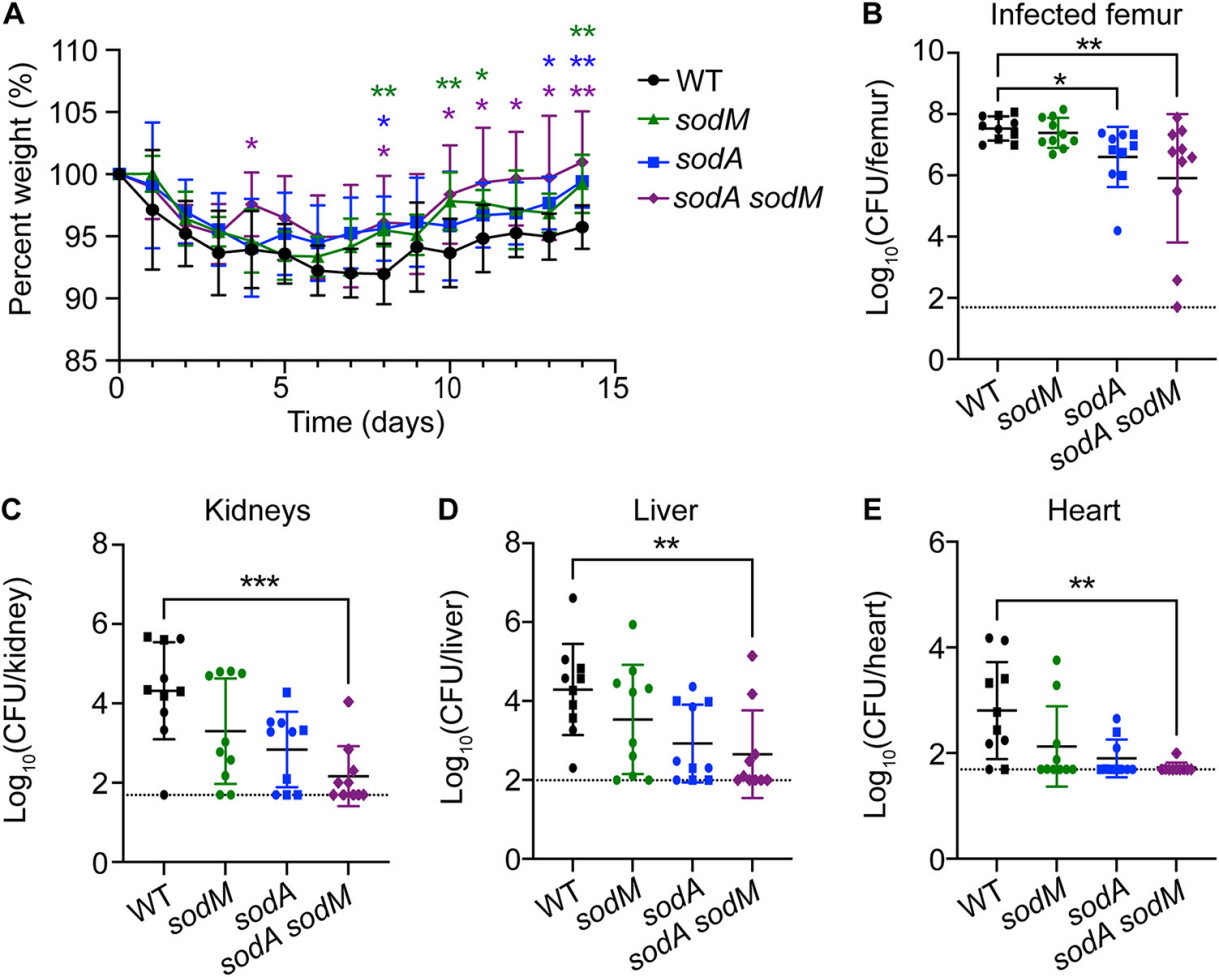
SodA is important for S. aureus survival during osteomyelitis in hyperglycemic mice. Eight-week-old male mice were treated with STZ intraperitoneally for 5 days. Ten days after the final injection, mice were infected with 1 × 10^5^ CFU of WT, *sodM*::erm, *sodA*::tet, and *sodA*::tet *sodM*::erm S. aureus strains via intraosseous injection. (A) Weights were recorded every 24 h and normalized to the starting weight of each animal on the day of infection (percent weight). (B to E) Mice were sacrificed at day 14 postinfection, and the bacterial burdens (CFU) were enumerated in infected femur (B), kidneys (C), liver (D), and heart (E). Different shapes indicate data collected from 3 distinct experiments (*n* = 10 mice per group). Dotted lines indicate limit of detection. Horizontal lines indicate means, and error bars represent SD. Significance was determined with two-way ANOVA and Dunnett’s multiple-comparison test (A) and Kruskal-Wallis with Dunn’s multiple-comparison test (B to E). *, *P* < 0.05; **, *P* < 0.01; ***, *P* < 0.001. All comparisons were made to the WT.

To validate the role of S. aureus SODs *in vivo*, we assessed the CFU burdens of WT, *sodA* mutant, *sodM* mutant, and *sodA sodM* mutant S. aureus in osteomyelitis monoinfections during hyperglycemia. Following STZ treatment, we inoculated mice with 1 × 10^5^ CFU of each mutant strain or WT. Over the course of the infection, mice were monitored for changes in weight. Mice infected with the *sodA*, *sodM*, and *sodA sodM* mutants had significantly less weight loss at multiple days postinfection compared to mice infected with WT (Fig. 6A). S. aureus burdens were lower in femurs of mice infected with the *sodA* and *sodA sodM* mutants than in those infected with WT at day 14 postinfection (Fig. 6B). Furthermore, the kidneys, liver, and heart from hyperglycemic mice infected with the *sodA sodM* mutant had lower S. aureus burdens than WT-infected mice (Fig. 6C to E). These data confirm the importance of SodA in promoting S. aureus osteomyelitis in the setting of hyperglycemia and validate the importance of SODs for bacterial dissemination.

**FIG 7:**
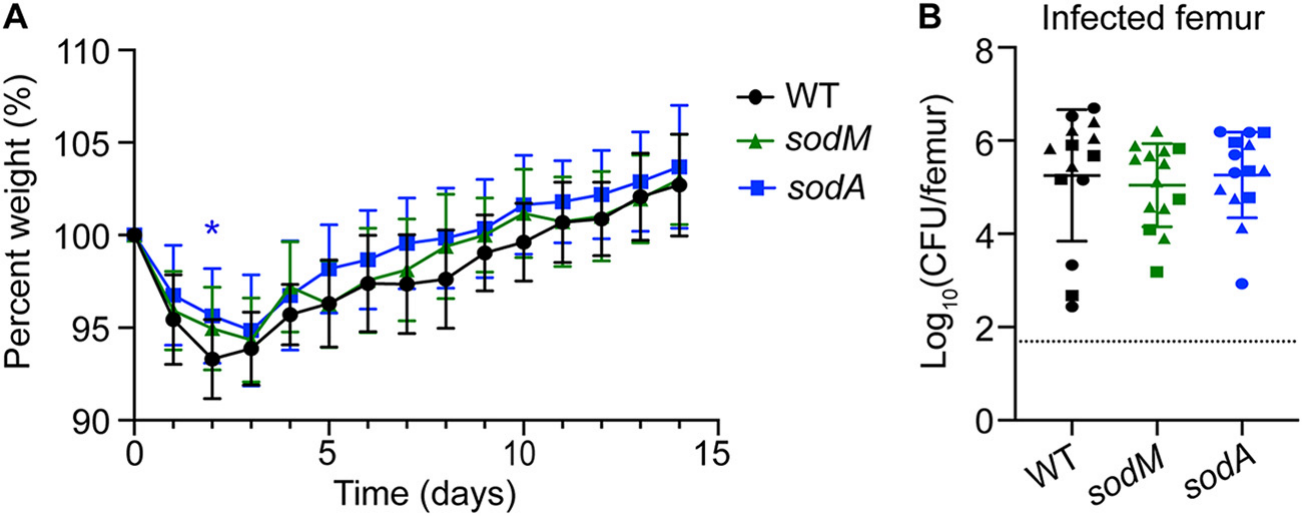
SodA is not necessary for S. aureus survival during osteomyelitis in euglycemic mice. Eight-week-old male mice were treated with sodium citrate (vehicle) intraperitoneally for 5 days. Ten days after the final injection, mice were infected with 1 × 10^5^ CFU of WT, *sodA*::tet, and *sodM*::erm S. aureus strains via intraosseous injection. (A) Weights were recorded every 24 h and normalized to the starting weight of each animal on the day of infection (percent weight). (B) Mice were sacrificed at day 14 postinfection, and the bacterial burdens (CFU) were enumerated in infected femurs. Different shapes indicate data collected from 3 distinct experiments (*n* = 14 mice per group). The dotted line indicates limit of detection. Horizontal lines indicate means, and error bars represent SD. Significance was determined with two-way ANOVA and Dunnett’s multiple-comparison test (A) and Kruskal-Wallis with Dunn’s multiple-comparison test (B). *, *P* < 0.05. All comparisons were made relative to WT.

To assess whether S. aureus SODs are essential for osteomyelitis in euglycemic mice, we inoculated vehicle-treated control mice with 1 × 10^5^ CFU of WT, *sodA* mutant, or *sodM* mutant S. aureus and measured bacterial burdens in the femur 14 days postinfection. The *sodA* mutant-infected mice exhibited a difference in percent starting weight compared to WT-infected mice at a single time point (day 2) postinfection (Fig. 7A). WT-, *sodA* mutant-, and *sodM* mutant-infected animals showed no differences in burdens in the infected femurs at day 14 postinfection (Fig. 7B). These data suggest that the fitness defect of the *sodA* mutant may be unique to hyperglycemic osteomyelitis.

## DISCUSSION

Using a chemically induced model of hyperglycemia, we discovered increased S. aureus burdens and significantly greater bone loss during hyperglycemic infection compared to euglycemic infection in a posttraumatic osteomyelitis model. We also discovered increased dissemination of S. aureus to other organs in hyperglycemic mice. The gene encoding SodA was found to be critical for S. aureus survival during hyperglycemic infection and for S. aureus survival *in vitro* in the presence of high glucose. These findings are consistent with prior clinical studies that correlated poor S. aureus infection outcomes in hyperglycemic individuals, both with and without diabetes (33, 34). This work further reveals a S. aureus virulence factor that contributes to increased osteomyelitis pathogenesis during hyperglycemia and supports the hypothesis that metabolic comorbidities shape the essential S. aureus genes required for invasive infection.

Multiple murine S. aureus infection models have identified increases in infection severity in the setting of hyperglycemia, including footpad infections in STZ-induced hyperglycemic mice (1, 35–37). More severe infections were also observed in STZ-induced hyperglycemic mice in an implant-related orthopedic infection model, as measured by S. aureus burdens, bone density, and biofilm formation (38). Models of type 2 diabetes likewise revealed increased osteomyelitis infection severity associated with hyperglycemia (39–41). Elevated S. aureus burdens have been observed in skin infection due to aberrant neutrophil chemotaxis, dysregulated abscess formation, and impaired wound healing during hyperglycemia (42, 43). Our study contributes to a greater understanding of how hyperglycemia influences S. aureus infection pathogenesis by revealing changes in bacterial survival during posttraumatic osteomyelitis as well as significantly greater infection-induced bone loss during hyperglycemia.

In prior studies, we identified genes involved in glucose metabolism and virulence as essential for S. aureus survival and disease pathogenesis *in vivo* during osteomyelitis (16, 18, 23). To identify genes that contribute to increased pathogenesis of S. aureus osteomyelitis during hyperglycemia, we conducted TnSeq, comparing S. aureus survival in hyperglycemic mice to that in euglycemic mice. Similar to our prior TnSeq study, genes involved in purine/pyrimidine and amino acid metabolism were identified as essential for S. aureus growth during osteomyelitis in hyperglycemic mice, including *carA*, *carB*, and *aptA*. Both *carA* and *aptA* are important for S. aureus survival in nitric oxide stress (19). Furthermore, *carA* is critical for S. aureus extracellular survival in the presence of high H_2_O_2_ (44). Purine and pyrimidine synthesis enables S. aureus to persist in the presence of hydroxyl radicals that oxidize base and ribose moieties of DNA, creating lesions that require repair (45). The gene *ccpA* was also essential for S. aureus growth during osteomyelitis in hyperglycemic mice, linking the abundance of glucose in tissues with transcriptional control of genes involved in gluconeogenesis, the tricarboxylic acid (TCA) cycle, and cell adherence and immune evasion (46–49).

TnSeq also revealed the importance of *sodA* for S. aureus survival during osteomyelitis in hyperglycemic mice. SodA has been implicated in S. aureus stress responses during aerobic metabolism and in the presence of ROS produced by innate immune cells (27, 32). Additionally, the expression of S. aureus *sodA* increases during skin infections in STZ-induced hyperglycemic mice (36). SodA catalyzes the detoxification of superoxide into H_2_O_2_, which is further broken down by catalase, and both SOD and catalase activities of bacteria have been correlated with virulence (27, 50–52). Production of superoxide is a known innate immune defense mechanism used to kill intracellular phagocytosed bacteria (53, 54). Previous publications revealed that neutrophils have a decreased capacity for respiratory burst and limited ROS production during hyperglycemic infection (5, 43, 55, 56). However, oxidative stress from altered metabolism in endothelial cells increases the amount of ROS in tissues of hyperglycemic patients (6, 57, 58). Superoxide can also be produced intrinsically by S. aureus in response to carbon overflow metabolism (28). Carbon overflow occurs when S. aureus breaks down glucose through glycolysis and results in acetate accumulation (28). The accumulation of acetate and NADH leads to a bottleneck in the electron transport chain that catalyzes reduction of oxygen, producing ROS (59). Based on these findings, we hypothesized that a *sodA* mutant would have a defect in survival in high glucose. We observed decreased S. aureus survival in glucose-rich media over 5 days in the absence of a functional *sodA* gene (60). These data are consistent with a model of hyperglycemic skin infection whereby dysfunctional phagocytes were observed to consume less glucose during hyperglycemia, thereby eliminating S. aureus competition for glucose and potentiating S. aureus virulence (35). These data suggest that the defect in survival of a *sodA* mutant may be related to intrinsic S. aureus ROS production during hyperglycemia.

The S. aureus genome includes two SOD genes, *sodA* and *sodM* (27, 29). The SODs are critical for S. aureus survival in the presence of superoxide during distinct phases of growth, with SodA functioning to detoxify ROS during exponential phase, while SodM has a greater role during stationary phase (29). Both S. aureus SODs can utilize manganese as a cofactor, while SodM can also use iron (31). The distinct characteristics of SodA and SodM may facilitate S. aureus survival in the presence of ROS in distinct tissues and/or nutritional microenvironments. Because SodA activity may be compensated by SodM, we decided to interrogate the roles of both genes in osteomyelitis during hyperglycemia. Mutating *sodA* or both *sodA* and *sodM* decreased S. aureus survival in hyperglycemic infection, while mutating *sodM* did not affect S. aureus survival, further supporting the importance of SodA in this model of infection. The greater attenuation of the *sodA sodM* mutant *in vivo* than mutation of *sodA* alone suggests potential redundancy of *sodM* in the absence of a functional *sodA* gene. In comparison, prior studies in euglycemic mice found that both *sodA* and *sodM* are essential for full S. aureus virulence following intravenous infection (61). These data suggest that there could be tissue and microenvironment-specific factors that influence the need for S. aureus SODs to support bacterial survival *in vivo*. We did not observe differences in the ability of WT and SOD mutants to survive osteomyelitis in euglycemic vehicle-treated mice. These findings are consistent with a skin abscess model of infection where there was no difference in abscess formation with a *sodA* mutant strain compared to WT in euglycemic mice (27).

There are some limitations to this study that should be considered. We chose to induce hyperglycemia with STZ to model increased blood glucose while minimizing confounding physiological changes associated with obesity, age, or the need for specialized housing (62). Limitations of this model include that STZ can be toxic to other organs, and the conclusions may not be generalizable to other models of hyperglycemia (62, 63). To model osteomyelitis, we used an established posttraumatic model, inoculating S. aureus directly into the femurs (18). This model does not effectively reproduce the characteristic clinical progression of contiguous wound dissemination that is commonly observed with diabetic foot wounds. The inoculum chosen in many *in vivo* experiments was 1 × 10^5^ CFU of S. aureus, which can lead to variability in the CFU quantified from infected organs and make calculating power difficult. This work also does not directly address how the host response was compromised during hyperglycemia, although multiple other studies using S. aureus infection models have identified changes in the immune response (2, 4, 5, 41, 42). Finally, TnSeq has inherent limitations, including the ability of compensatory mutants to rescue the growth of transposon mutants via nutrient sharing or other community interactions.

Future studies should include other models of osteomyelitis, such as footpad infections, to observe whether osteomyelitis infection dynamics are recapitulated in contiguous wound infection models. Additionally, studies are needed to assess which host processes influence the role of SodA in infection. Infections with WT and *sodA* mutant S. aureus in euglycemic and hyperglycemic mice with the inability to undergo respiratory burst may reveal the contributions of host-derived ROS versus the contribution of circulating blood glucose concentration. Furthermore, the conditions in which SodA is beneficial for S. aureus survival should be assessed. For example, a longitudinal study measuring ROS paired with a *sodA* luminescent or fluorescent reporter could reveal the context in which SodA influences S. aureus survival. Additionally, S. aureus glucose transporter mutants can be used to assess the ability of S. aureus to survive under hyperglycemic conditions when it does not have the ability to use exogenous glucose (64). Measuring changes in glucose and glucose-derived metabolites within the microenvironment of infected femurs in hyperglycemic mice is also an important future direction.

Taken together, the data in this report reveal that both acute hyperglycemia and chronic hyperglycemia increase S. aureus infectious burden, dissemination, and bone destruction during osteomyelitis. We identified 71 genes that are uniquely essential for S. aureus growth during osteomyelitis in hyperglycemic mice compared to euglycemic osteomyelitis. Of these genes, *sodA* was further studied due to its role in detoxifying ROS, a by-product of glucose carbon overflow metabolism. The results of this study highlight a bacterial virulence gene that contributes to exacerbated infection during hyperglycemia and provide a strong rationale for continued investigation into mechanisms of enhanced musculoskeletal disease in the context of hyperglycemia.

## MATERIALS AND METHODS

### Bacterial strains and culture conditions.

Unless otherwise stated, experiments were performed with S. aureus USA300 lineage strain AH1263, which served as wild type (WT). AH1263 is an erythromycin- and tetracycline-sensitive derivative of strain LAC and is representative of one of the most common S. aureus lineages isolated from musculoskeletal infections (65). *sodA*::tet (referred to as *sodA*), *sodM*::erm (referred to as *sodM*), and *sodA*::tet *sodM*::erm (referred to as *sodA sodM*) strains in the AH1263 background were created via phi-85-mediated phage transduction of *sodA*::tet and *sodM*::erm from the Newman background (29, 31). The *sodA* complementation construct was created by amplifying *sodA* and its endogenous promoter with primer sequences CTAGCTCTAGATGAGATTTATGCACATTTGGTCA and CTAGCGGTACCTTTATTTTGTTGCATTATATAATTCG. The *sodA* sequence was ligated into pJC1111 and integrated into the chromosome at attachment (*attC*) sites, as previously described (66). All bacterial cultures were grown overnight in 5 mL of tryptic soy broth (TSB) at 37°C with shaking at 180 rpm, except as otherwise noted. Erythromycin (10 μg/mL), tetracycline (2 μg/mL), or cadmium chloride (0.1 mM) was added to cultures with strains possessing the corresponding antibiotic resistance markers.

### Murine model of osteomyelitis.

All animal experiments were reviewed and approved by the Institutional Animal Care and Use Committee at Vanderbilt University Medical Center and performed in accordance with NIH guidelines, the Animal Welfare Act, and U.S. federal law. Six- to 8-week-old male C57BL/6J mice were obtained from Jackson Laboratories (stock number 000664) and intraperitoneally injected daily with 200 μL of 0.1 mM sodium citrate (vehicle) or 40 to 60 mg/kg of body weight streptozotocin (STZ) in 200 μL of 0.1 mM sodium citrate for 5 days to induce hyperglycemia. Acute hyperglycemia infections were performed 10 days after the final intraperitoneal STZ or sodium citrate injection, and chronic hyperglycemia infections were performed 30 days the final intraperitoneal STZ or sodium citrate injection. Blood glucose concentrations were quantified from a tail vein bleed immediately prior to inoculation (day 0) and on the day of sacrifice (day 14). STZ-treated mice below the hyperglycemic threshold of 250 mg/dL were removed from the study, except as otherwise noted. Osteomyelitis was induced with ~1 × 10^6^ or ~1 × 10^5^ CFU in 2 μL via intraosseous injection into the femurs, as previously reported (18). Mice were weighed daily and monitored for disease progression. Fourteen days postinfection, mice were sacrificed, and the infected femurs were either homogenized for CFU enumeration or fixed for μCT (see below). For CFU enumeration, infected femur, contralateral femur, kidneys, liver, and heart were homogenized in cell lytic buffer (Sigma) and plated on tryptic soy agar (TSA). Limits of detection based on volume of homogenate plated were 49 CFU per femur, heart, and kidneys and 99 CFU per liver.

### Microcomputed tomography analysis of femurs.

Infected and contralateral femurs were harvested from mice at day 0 for baseline μCT measurements and at day 14 postinfection. Femurs were fixed in 10% neutral buffered formalin for 2 days and then moved to 70% ethanol and stored at 4°C. Fixed femurs were scanned with a μCT50 Scanco instrument (Scanco Medical, Switzerland) and analyzed with μCT V6.3-4 software (Scanco USA, Inc.). The diaphysis and distal epiphysis of each femur were imaged with 10.0-μm voxel size at 70 kV and 200 μA with an integration time of 350 ms in 10.24-mm view. A total of 1,088 slices were obtained to include the diaphysis surrounding the cortical defect formed during inoculation as well as trabecular bone in the distal femur. Three-dimensional reconstructions were analyzed to quantify cortical bone destruction surrounding the inoculation site (in cubic millimeters). Trabecular bone volume per total volume (percent), trabecular number (1/millimeter), trabecular thickness (in millimeters), and trabecular spacing (in millimeters) were quantified as previously described (18, 25).

### Bone histology.

After μCT imaging, femurs were decalcified in 20% EDTA for 4 days at 4°C. Decalcified femurs were processed into paraffin, embedded, and sectioned through the infectious nidus and bone marrow cavity at a thickness of 4 μm with a Leica RM2255 microtome. Sections were stained with hematoxylin and eosin (H&E). A Leica SCN400 slide scanner was used to scan stained femur sections at 20×, and images were uploaded to and analyzed within the Digital Slide Archive (Vanderbilt University Medical Center).

### Transposon sequencing analysis of experimental osteomyelitis.

USA300 LAC transposon library aliquots were obtained and expanded in 10 mL of BHI in 50 mL Erlenmeyer flasks loosely covered with foil for 6 h at 37°C with shaking at 180 rpm (19, 67). The expanded library was collected by centrifugation, aliquoted for individual experiments, and thawed on ice as needed, as previously described (67). Briefly, library aliquots were centrifuged at 200 × *g* for 8 min at 4°C and resuspended in cold, sterile phosphate-buffered saline (PBS) to achieve an inoculum concentration of ~2.5 × 10^9^ CFU/mL. A total of 2 μL of inoculum was delivered (final concentration of ~5 × 10^6^ CFU) via intraosseous injection into the femurs of C57BL/6J male mice treated with vehicle or STZ, as described above. Mice were sacrificed at day 4 based on prior studies (23). Femurs were homogenized in 500 μL of cold, sterile PBS. A total of 150 μL of bone homogenate from two mice was pooled in 4.7 mL of BHI in 50 mL Erlenmeyer flasks loosely covered with foil for a 2 h outgrowth step at 37°C with shaking at 180 rpm. Following outgrowth, host debris was allowed to settle to the bottom of the culture, and the top fraction was transferred to a conical on ice. The top fraction was pelleted at 8,000 × *g* for 8 min at 4°C, resuspended in an equal volume of 20% glycerol BHI, and stored at −80°C. In parallel, 2 μL of prepared inoculum was inoculated into 50 mL of BHI in a 250 mL Erlenmeyer flask to serve as an *in vitro* comparator. After 24 h of growth at 37°C with shaking at 180 rpm and being covered loosely with foil, the cultures were pelleted at 8,000 × *g* for 8 min at 4°C, supernatant was discarded, and samples were stored at −80°C.

### Library preparation and analysis of transposon sequencing.

Genomic DNA was isolated with a phenol-chloroform-isoamyl alcohol protocol as described previously (67). The DNA was then sheared to ~350 bp by sonication using a Covaris LE220 instrument. Libraries were prepared for sequencing with the homopolymer tail-mediated ligation PCR technique (68). Terminal deoxytransferase was used to generate a poly(C)-tailed sequence on the 3′ end of the DNA fragments. The transposon junctions were amplified with two rounds of nested PCR and multiplexed with 8 bp indexing primers. The indexed DNA fragments were sequenced on an Illumina Hi-Seq 2500 (Tufts University Core Facility). Reads were trimmed, filtered for quality, and mapped to S. aureus FPR3757 (GenBank accession number NC_007793). A Dval score was assigned to each gene based on the number of reads within a given gene in a sample divided by the predicted number of reads for the gene considering its size and total sequencing reads for the given sample. Genes with a Dval between 0.1 and 0.01 were considered compromised under each condition, and genes with a Dval of ≤0.01 were considered essential.

### Comparative S. aureus survival analysis *in vitro* at different concentrations of glucose.

Overnight cultures of WT and mutant strains were washed in PBS and back diluted 1:1,000 into 10 mL of TSB (containing 250 mg/dL of glucose) with and without 500 mg/dL of added glucose in 50 mL Erlenmeyer flasks. For some experiments, TSB was adjusted to a pH of 4.5, 5.5, 6.5, or 7.5 with HCl and NaOH. Cultures were grown at 37°C with shaking at 180 rpm either loosely covered with foil (aerobic) or plugged with a rubber stopper (microaerobic). Viable CFU were measured by serially diluting cultures and plating on TSA at the indicated time points. Growth was reported as log_10_ CFU per milliliter compared to CFU enumerated at 0 h. pH was measured over the course of experiments with pH test strips (Fisher Scientific; 13-640-516).

### Graphical and statistical analyses.

Statistical analyses were performed with Prism 9.4.1 (GraphPad Software). Data were checked for normality prior to statistical analysis. In comparisons of two groups, including the comparisons made for CFU burdens and μCT parameters, Mann-Whitney tests were used. To assess the importance of SOD genes for S. aureus survival over time *in vitro*, two-way analyses of variance (ANOVA) with *post hoc* Dunnett’s multiple-comparison tests were used to compare the influence of the genotype at each time point. Two-way ANOVA with *post hoc* Dunnett’s multiple-comparison tests were also used to compare the percent weight of animals infected with different S. aureus strains or subjected to different treatments (STZ or vehicle) over 14 days. To measure changes in S. aureus survival *in vivo* in experiments with 3 or more bacterial strains, one-way ANOVA with Kruskal-Wallis and Dunn’s multiple comparisons were used to compare mutant strain survival to WT survival due to the non-Gaussian distribution of the data. Changes in S. aureus bacterial burden at day 14 in vehicle-treated, STZ-treated and not hyperglycemic, and STZ-treated hyperglycemic mice were compared with one-way ANOVA and Tukey’s multiple-comparison test.

## References

[1] Brandt SL, Wang S, Dejani NN, Klopfenstein N, Winfree S, Filgueiras L, McCarthy BP, Territo PR, Serezani CH. 2018. Excessive localized leukotriene B4 levels dictate poor skin host defense in diabetic mice. JCI Insight 3:e120220. 10.1172/jci.insight.120220. 30185672 PMC6171805

[2] Dejani NN, Brandt SL, Pineros A, Glosson-Byers NL, Wang S, Son YM, Medeiros AI, Serezani CH. 2016. Topical prostaglandin E analog restores defective dendritic cell-mediated Th17 host defense against methicillin-resistant *Staphylococcus aureus* in the skin of diabetic mice. Diabetes 65:3718–3729. 10.2337/db16-0565. 27605625 PMC5127243

[3] Brandt SL, Klopfenstein N, Wang S, Winfree S, McCarthy BP, Territo PR, Miller L, Serezani CH. 2018. Macrophage-derived LTB4 promotes abscess formation and clearance of *Staphylococcus aureus* skin infection in mice. PLoS Pathog 14:e1007244. 10.1371/journal.ppat.1007244. 30102746 PMC6107286

[4] Filgueiras RL, Brandt SL, de Oliveira Ramalho TR, Jancar S, Serezani CH. 2017. Imbalance between HDAC and HAT activities drives aberrant STAT1/MyD88 expression in macrophages from type 1 diabetic mice. J Diabetes Complications 31:334–339. 10.1016/j.jdiacomp.2016.08.001. 27623388 PMC5296405

[5] Rich J, Lee JC. 2005. The pathogenesis of *Staphylococcus aureus* infection in the diabetic NOD mouse. Diabetes 54:2904–2910. 10.2337/diabetes.54.10.2904. 16186391

[6] Giacco F, Brownlee M. 2010. Oxidative stress and diabetic complications. Circ Res 107:579–591. 10.1161/CIRCRESAHA.110.223545. 21030723 PMC2996922

[7] Filgueiras LR, Brandt SL, Wang S, Wang Z, Morris DL, Evans-Molina C, Mirmira RG, Jancar S, Serezani CH. 2015. Leukotriene B4-mediated sterile inflammation promotes susceptibility to sepsis in a mouse model of type 1 diabetes. Sci Signal 8:ra10. 10.1126/scisignal.2005568. 25628460 PMC4356178

[8] Liemburg-Apers DC, Willems PHGM, Koopman WJH, Grefte S. 2015. Interactions between mitochondrial reactive oxygen species and cellular glucose metabolism. Arch Toxicol 89:1209–1226. 10.1007/s00204-015-1520-y. 26047665 PMC4508370

[9] Muller LMAJ, Gorter KJ, Hak E, Goudzwaard WL, Schellevis FG, Hoepelman AIM, Rutten GEHM. 2005. Increased risk of common infections in patients with type 1 and type 2 diabetes mellitus. Clin Infect Dis 41:281–288. 10.1086/431587. 16007521

[10] Evans JL, Goldfine ID, Maddux BA, Grodsky GM. 2003. Are oxidative stress–activated signaling pathways mediators of insulin resistance and β-cell dysfunction? Diabetes 52:1–8. 10.2337/diabetes.52.1.1. 12502486

[11] Cassat JE, Gimza BD. 2021. Mechanisms of antibiotic failure during *Staphylococcus aureus* osteomyelitis. Front Immunol 12:638085. 10.3389/fimmu.2021.638085. 33643322 PMC7907425

[12] Berendt T, Byren I. 2004. Bone and joint infection. Clin Med 4:510–518. 10.1016/j.mpmed.2017.08.003. PMC4951986 15656476

[13] Baeta L-C. 2017. Diabetes, bone and glucose-lowering agents: basic biology. Diabetologia 60:1163–1169. 10.1007/S00125-017-4269-4. 28434032 PMC5487688

[14] Jiao H, Xiao E, Graves DT. 2015. Diabetes and its effect on bone and fracture healing. Curr Osteoporos Rep 13:327–335. 10.1007/s11914-015-0286-8. 26254939 PMC4692363

[15] Vitko NP, Spahich NA, Richardson AR. 2015. Glycolytic dependency of high-level nitric oxide resistance and virulence in *Staphylococcus aureus*. mBio 6:e00045-15. 10.1128/mBio.00045-15. 25852157 PMC4453550

[16] Potter AD, Butrico CE, Ford CA, Curry JM, Trenary IA, Tummarakota SS, Hendrix AS, Young JD, Cassat JE. 2020. Host nutrient milieu drives an essential role for aspartate biosynthesis during invasive *Staphylococcus aureus* infection. Proc Natl Acad Sci USA 117:12394–12401. 10.1073/pnas.1922211117. 32414924 PMC7275739

[17] Butrico CE, Cassat JE. 2020. Quorum sensing and toxin production in *Staphylococcus aureus* osteomyelitis: pathogenesis and paradox. Toxins 12:516. 10.3390/toxins12080516. 32806558 PMC7471978

[18] Cassat JE, Hammer ND, Campbell JP, Benson MA, Perrien DS, Mrak LN, Smeltzer MS, Torres VJ, Skaar EP. 2013. A secreted bacterial protease tailors the *Staphylococcus aureus* virulence repertoire to modulate bone remodeling during osteomyelitis. Cell Host Microbe 13:759–772. 10.1016/j.chom.2013.05.003. 23768499 PMC3721972

[19] Grosser MR, Paluscio E, Thurlow LR, Dillon MM, Cooper VS, Kawula TH, Richardson AR. 2018. Genetic requirements for *Staphylococcus aureus* nitric oxide resistance and virulence. PLoS Pathog 14:e1006907. 10.1371/journal.ppat.1006907. 29554137 PMC5884563

[20] Arora S, Ojha KS, Vohora D. 2009. Characterisation of streptozotocin induced diabetes mellitus in Swiss albino mice. Glob J Pharmacol 3:81–84.

[21] Rossini AA, Williams RM, Appel MC, Like AA, Joslin EP. 1978. Sex differences in the multiple-dose streptozotocin model of diabetes. Endocrinology 103:1518–1520. 10.1210/endo-103-4-1518. 154403

[22] Leiter EH. 1982. Multiple low-dose streptozotocin-induced hyperglycemia and insulitis in C57BL mice: influence of inbred background, sex, and thymus. Proc Natl Acad Sci USA 79:630–634. 10.1073/pnas.79.2.630. 6210909 PMC345800

[23] Wilde AD, Snyder DJ, Putnam NE, Valentino MD, Hammer ND, Lonergan ZR, Hinger SA, Aysanoa EE, Blanchard C, Dunman PM, Wasserman GA, Chen J, Shopsin B, Gilmore MS, Skaar EP, Cassat JE. 2015. Bacterial hypoxic responses revealed as critical determinants of the host-pathogen outcome by TnSeq analysis of *Staphylococcus aureus* invasive infection. PLoS Pathog 11:e1005341. 10.1371/journal.ppat.1005341. 26684646 PMC4684308

[24] Klopfenstein N, Brandt SL, Castellanos S, Gunzer M, Blackman A, Serezani CH. 2021. SOCS-1 inhibition of type I interferon restrains *Staphylococcus aureus* skin host defense. PLoS Pathog 17:e1009387. 10.1371/journal.ppat.1009387. 33690673 PMC7984627

[25] Putnam NE, Fulbright LE, Curry JM, Ford CA, Petronglo JR, Hendrix AS, Cassat JE. 2019. MyD88 and IL-1R signaling drive antibacterial immunity and osteoclast-driven bone loss during *Staphylococcus aureus* osteomyelitis. PLoS Pathog 15:e1007744. 10.1371/journal.ppat.1007744. 30978245 PMC6481883

[26] Napoli N, Chandran M, Pierroz DD, Abrahamsen B, Schwartz A, Ferrari SL, IOF Bone and Diabetes Working Group. 2017. Mechanisms of diabetes mellitus-induced bone fragility. Nat Rev Endocrinol 13:208–219. 10.1038/nrendo.2016.153. 27658727

[27] Clements MO, Watson SP, Foster SJ. 1999. Characterization of the major superoxide dismutase of *Staphylococcus aureus* and its role in starvation survival, stress resistance, and pathogenicity. J Bacteriol 181:3898–3903. 10.1128/JB.181.13.3898-3903.1999. 10383955 PMC93877

[28] Thomas VC, Sadykov MR, Chaudhari SS, Jones J, Endres JL, Widhelm TJ, Ahn J-S, Jawa RS, Zimmerman MC, Bayles KW. 2014. A central role for carbon-overflow pathways in the modulation of bacterial cell death. PLoS Pathog 10:e1004205. 10.1371/journal.ppat.1004205. 24945831 PMC4063974

[29] Valderas MW, Hart ME. 2001. Identification and characterization of a second superoxide dismutase gene (*sodM*) from *Staphylococcus aureus*. J Bacteriol 183:3399–3407. 10.1128/JB.183.11.3399-3407.2001. 11344148 PMC99638

[30] Ballal A, Manna AC. 2009. Regulation of superoxide dismutase (*sod*) genes by SarA in *Staphylococcus aureus*. J Bacteriol 191:3301–3310. 10.1128/JB.01496-08. 19286803 PMC2687179

[31] Garcia YM, Barwinska-Sendra A, Tarrant E, Skaar EP, Waldron KJ, Kehl-Fie TE. 2017. A superoxide dismutase capable of functioning with iron or manganese promotes the resistance of *Staphylococcus aureus* to calprotectin and nutritional immunity. PLoS Pathog 13:e1006125. 10.1371/journal.ppat.1006125. 28103306 PMC5245786

[32] Karavolos MH, Horsburgh M, Ingham E, Foster SJ. 2003. Role and regulation of the superoxide dismutases of *Staphylococcus aureus*. Microbiology (Reading) 149:2749–2758. 10.1099/mic.0.26353-0. 14523108

[33] Forsblom E, Ruotsalainen E, Järvinen A. 2017. Prognostic impact of hyperglycemia at onset of methicillin-sensitive *Staphylococcus aureus* bacteraemia. Eur J Clin Microbiol Infect Dis 36:1405–1413. 10.1007/s10096-017-2946-3. 28265815

[34] Bader MS. 2007. Hyperglycemia and mortality in elderly patients with *Staphylococcus aureus* bacteremia. South Med J 100:252–256. 10.1097/01.smj.0000257383.66288.68. 17396726

[35] Thurlow LR, Stephens AC, Hurley KE, Richardson AR. 2020. Lack of nutritional immunity in diabetic skin infections promotes *Staphylococcus aureus* virulence. Sci Adv 6:eabc5569. 10.1126/sciadv.abc5569. 33188027 PMC7673755

[36] Jacquet R, LaBauve AE, Akoolo L, Patel S, Alqarzaee AA, Wong Fok Lung T, Poorey K, Stinear TP, Thomas VC, Meagher RJ, Parker D. 2019. Dual gene expression analysis identifies factors associated with *Staphylococcus aureus* virulence in diabetic mice. Infect Immun 87:e00163-19. 10.1128/IAI.00163-19. 30833333 PMC6479027

[37] Tuchscherr L, Korpos E, van de Vyver H, Findeisen C, Kherkheulidze S, Siegmund A, Deinhardt-Emmer S, Bach O, Rindert M, Mellmann A, Sunderkotter C, Peters G, Sorokin L, Loffler B. 2018. *Staphylococcus aureus* requires less virulence to establish an infection in diabetic hosts. Int J Med Microbiol 308:761–769. 10.1016/j.ijmm.2018.05.004. 29843979

[38] Lovati AB, Drago L, Monti L, De Vecchi E, Previdi S, Banfi G, Romano CL. 2013. Diabetic mouse model of orthopaedic implant-related *Staphylococcus aureus* infection. PLoS One 8:e67628. 10.1371/journal.pone.0067628. 23818985 PMC3688606

[39] Farnsworth CW, Schott EM, Benvie AM, Zukoski J, Kates SL, Schwarz EM, Gill SR, Zuscik MJ, Mooney RA. 2018. Obesity/type 2 diabetes increases inflammation, periosteal reactive bone formation, and osteolysis during *Staphylococcus aureus* implant-associated bone infection. J Orthop Res 36:1614–1623. 10.1002/jor.23831. 29227579 PMC5995608

[40] Farnsworth CW, Schott EM, Jensen SE, Zukoski J, Benvie AM, Refaai MA, Kates SL, Schwarz EM, Zuscik MJ, Gill SR, Mooney RA. 2017. Adaptive upregulation of clumping factor A (ClfA) by *Staphylococcus aureus* in the obese, type 2 diabetic host mediates increased virulence. Infect Immun 85:e01005-16. 10.1128/IAI.01005-16. 28320836 PMC5442639

[41] Farnsworth CW, Shehatou CT, Maynard R, Nishitani K, Kates SL, Zuscik MJ, Schwarz EM, Daiss JL, Mooney RA. 2015. A humoral immune defect distinguishes the response to *Staphylococcus aureus* infections in mice with obesity and type 2 diabetes from that in mice with type 1 diabetes. Infect Immun 83:2264–2274. 10.1128/IAI.03074-14. 25802056 PMC4432732

[42] Brandt SL, Serezani CH. 2017. Too much of a good thing: how modulating LTB_4_ actions restore host defense in homeostasis or disease. Semin Immunol 33:37–43. 10.1016/j.smim.2017.08.006. 29042027 PMC5679129

[43] Wong SL, Demers M, Martinod K, Gallant M, Wang Y, Goldfine AB, Kahn CR, Wagner DD. 2015. Diabetes primes neutrophils to undergo NETosis, which impairs wound healing. Nat Med 21:815–819. 10.1038/nm.3887. 26076037 PMC4631120

[44] Buvelot H, Roth M, Jaquet V, Lozkhin A, Renzoni A, Bonetti E-J, Gaia N, Laumay F, Mollin M, Stasia M-J, Schrenzel J, Francois P, Krause K-H. 2021. Hydrogen peroxide affects growth of *S. aureus* through downregulation of genes involved in pyrimidine biosynthesis. Front Immunol 12:673985. 10.3389/fimmu.2021.673985. 34557184 PMC8454235

[45] Imlay JA. 2013. The molecular mechanisms and physiological consequences of oxidative stress: lessons from a model bacterium. Nat Rev Microbiol 11:443–454. 10.1038/nrmicro3032. 23712352 PMC4018742

[46] Seidl K, Stucki M, Ruegg M, Goerke C, Wolz C, Harris L, Berger-Bachi B, Bischoff M. 2006. *Staphylococcus aureus* CcpA affects virulence determinant production and antibiotic resistance. Antimicrob Agents Chemother 50:1183–1194. 10.1128/AAC.50.4.1183-1194.2006. 16569828 PMC1426959

[47] Nuxoll AS, Halouska SM, Sadykov MR, Hanke ML, Bayles KW, Kielian T, Powers R, Fey PD. 2012. CcpA regulates arginine biosynthesis in *Staphylococcus aureus* through repression of proline catabolism. PLoS Pathog 8:e1003033. 10.1371/journal.ppat.1003033. 23209408 PMC3510247

[48] Seidl K, Muller S, Francois P, Kriebitzsch C, Schrenzel J, Engelmann S, Bischoff M, Berger-Bach B. 2009. Effect of a glucose impulse on the CcpA regulon in *Staphylococcus aureus*. BMC Microbiol 9:95. 10.1186/1471-2180-9-95. 19450265 PMC2697999

[49] Richardson AR, Somerville GA, Sonenshein AL. 2015. Regulating the intersection of metabolism and pathogenesis in gram-positive bacteria. Microbiol Spectr 3:MBP-0004-2014. 10.1128/microbiolspec.MBP-0004-2014. PMC4540601 26185086

[50] Tally FP, Goldin BR, Jacobus NV, Gorbach SL. 1977. Superoxide dismutase in anaerobic bacteria of clinical significance. Infect Immun 16:20–25. 10.1128/iai.16.1.20-25.1977. 326669 PMC421481

[51] Mandell GL. 1975. Catalase, superoxide dismutase, and virulence of *Staphylococcus aureus*: *in vitro* and *in vivo* studies with emphasis on staphylococcal-leukocyte interaction. J Clin Invest 55:561–566. 10.1172/JCI107963. 1117067 PMC301784

[52] Farrant JL, Sansone A, Canvin JR, Pallen MJ, Langford PR, Wallis TS, Dougan G, Kroll JS. 1997. Bacterial copper- and zinc-cofactored superoxide dismutase contributes to the pathogenesis of systemic salmonellosis. Mol Microbiol 25:785–796. 10.1046/j.1365-2958.1997.5151877.x. 9379906

[53] Ganz T. 1999. Oxygen-independent microbicidal mechanisms of phagocytes. Proc Assoc Am Physicians 111:390–395. 10.1111/paa.1999.111.5.390. 10519158

[54] Segal AW. 1989. The electron transport chain of the microbicidal oxidase of phagocytic cells and its involvement in the molecular pathology of chronic granulomatous disease. J Clin Invest 83:1785–1793. 10.1172/JCI114083. 2656760 PMC303897

[55] Marhoffer W, Stein M, Schleinkofer L, Federlin K. 1993. Evidence of *ex vivo* and *in vitro* impaired neutrophil oxidative burst and phagocytic capacity in type 1 diabetes mellitus. Diabetes Res Clin Pract 19:183–188. 10.1016/0168-8227(93)90112-i. 8319516

[56] Marhoffer W, Stein M, Maeser E, Federlin K. 1992. Impairment of polymorphonuclear leukocyte function and metabolic control of diabetes. Diabetes Care 15:256–260. 10.2337/diacare.15.2.256. 1547682

[57] Yan LJ. 2014. Pathogenesis of chronic hyperglycemia: from reductive stress to oxidative stress. J Diabetes Res 2014:137919. 10.1155/2014/137919. 25019091 PMC4082845

[58] Volpe CMO, Villar-Delfino PH, dos Anjos PMF, Nogueira-Machado JA. 2018. Cellular death, reactive oxygen species (ROS) and diabetic complications. Cell Death Dis 9:119. 10.1038/s41419-017-0135-z. 29371661 PMC5833737

[59] Lemire J, Alhasawi A, Appanna VP, Tharmalingam S, Appanna VD. 2017. Metabolic defence against oxidative stress: the road less travelled so far. J Appl Microbiol 123:798–809. 10.1111/jam.13509. 28609580

[60] Thomas VC, Chaudhari SS, Jones J, Zimmerman MC, Bayles KW. 2015. Electron paramagnetic resonance (EPR) spectroscopy to detect reactive oxygen species in *Staphylococcus aureus*. Bio Protoc 5:e1586. 10.21769/bioprotoc.1589. PMC4863951 27182534

[61] Kehl-Fie TE, Chitayat S, Hood MI, Damo S, Restrepo N, Garcia C, Munro KA, Chazin WJ, Skaar EP. 2011. Nutrient metal sequestration by calprotectin inhibits bacterial superoxide defense, enhancing neutrophil killing of *Staphylococcus aureus*. Cell Host Microbe 10:158–164. 10.1016/j.chom.2011.07.004. 21843872 PMC3157011

[62] King AJF. 2012. The use of animal models in diabetes research. Br J Pharmacol 166:877–894. 10.1111/j.1476-5381.2012.01911.x. 22352879 PMC3417415

[63] Lee JH, Yang SH, Oh JM, Lee MG. 2010. Pharmacokinetics of drugs in rats with diabetes mellitus induced by alloxan or streptozocin: comparison with those in patients with type I diabetes mellitus. J Pharm Pharmacol 62:1–23. 10.1211/jpp.62.01.0001. 20722995

[64] Vitko NP, Grosser MR, Khatri D, Lance TR, Richardson AR. 2016. Expanded glucose import capability affords *Staphylococcus aureus* optimized glycolytic flux during infection. mBio 7:e00296-16. 10.1128/mBio.00296-16. 27329749 PMC4916373

[65] Carrillo-Marquez MA, Hulten KG, Hammerman W, Mason EO, Kaplan SL. 2009. USA300 is the predominant genotype causing *Staphylococcus aureus* septic arthritis in children. Pediatr Infect Dis J 28:1076–1080. 10.1097/INF.0b013e3181adbcfe. 19820424

[66] Fey PD, Endres JL, Yajjala VK, Widhelm TJ, Boissy RJ, Bose JL, Bayles KW. 2013. A genetic resource for rapid and comprehensive phenotype screening of nonessential *Staphylococcus aureus* genes. mBio 4:e00537-12. 10.1128/mBio.00537-12. 23404398 PMC3573662

[67] Peek CT, Ibberson CB, Cassat JE. 2020. Identification of virulence determinants during host-pathogen interaction using Tn-Seq technology. Methods Mol Biol 2069:155–175. 10.1007/978-1-4939-9849-4. 31523773

[68] Lazinski EW, Camilli A. 2013. Homopolymer tail-mediated ligation PCR: a streamlined and highly efficient method for DNA cloning and library construction. Biotechniques 54:25–34. 10.2144/000113981. 23311318 PMC3605734

