# Supplementary material for: Hyperglycemia Increases Severity of Staphylococcus aureus Osteomyelitis and Influences Bacterial Genes Required for Survival in Bone
Source: Infect Immun. 2023 Mar 6;91(4):e00529-22. doi: 10.1128/iai.00529-22 (PMC10112148; doi:10.1128/iai.00529-22)
Supplement: Supplemental file 1 — Fig. S1 to S8 and Tables S1 to S2. Download iai.00529-22-s0001.pdf, PDF file, 8.4 MB [file iai.00529-22-s0001.pdf]

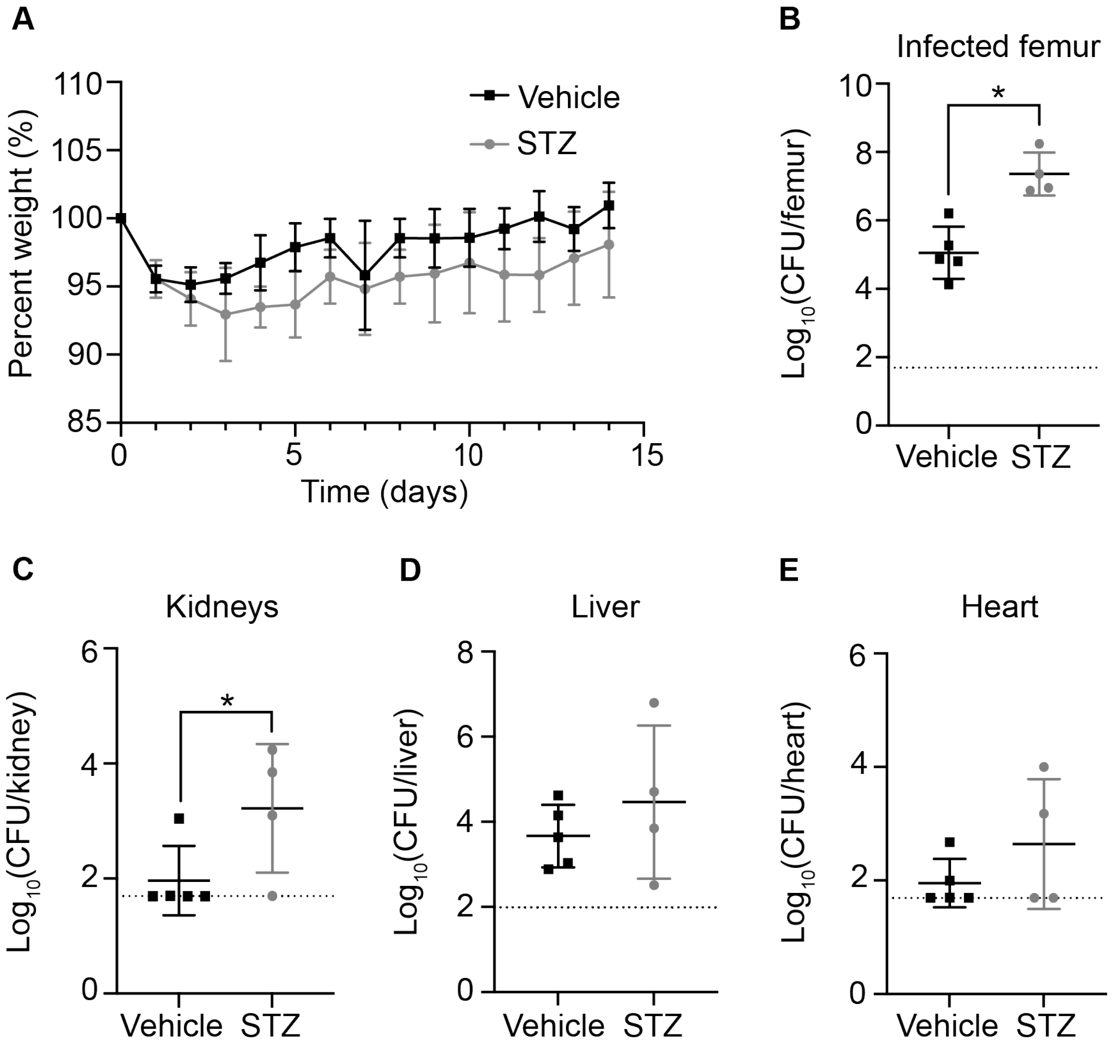

Figure S1. *S. aureus* survival during osteomyelitis increases during acute hyperglycemia with a lower inoculum. Eight-week old male mice were treated with sodium citrate (vehicle) or streptozotocin (STZ) intraperitoneally for 5 days. 10 days after the final injection, mice were infected with $1x10^5$ CFU of WT *S. aureus* via intraosseous injection. (A) Weights were recorded every 24 hrs and normalized to the starting weight of each animal on the day of infection (percent weight). Mice were sacrificed at day 14 post-infection, and the bacterial burdens (CFU) were enumerated in (B) infected femur, (C) kidneys, (D) liver, and (E) heart. One experiment was conducted with n = 5 mice per group. Dotted lines indicate limit of detection. Horizontal lines indicate mean, and error bars represent SD. Significance determined multiple paired t-tests (A) and Mann-Whitney test (B-E). *p<0.01.

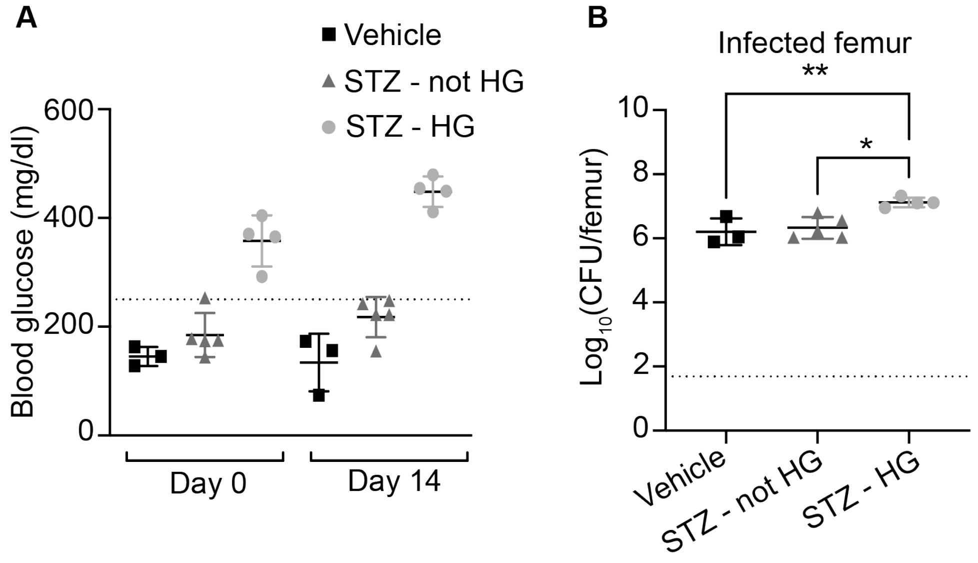

Figure S2. *S. aureus* bacterial burdens are higher in STZ-treated hyperglycemic mice compared to euglycemic STZ- or vehicle-treated mice during osteomyelitis. Eight-week old male mice were treated with sodium citrate (vehicle) or streptozotocin (STZ) intraperitoneally for 5 days. 10 days after the final injection, mice were infected with $1x10^6$ CFU of WT *S. aureus* via intraosseous injection. (A) Blood glucose concentration was quantified from the tail vein on the day of infection (day 0) and the day of sacrifice (day 14). (B) Mice were sacrificed at day 14 post-infection, and the bacterial burdens (CFU) were enumerated in infected femurs. One experiment was conducted with n = 3 mice for vehicle group, n = 5 mice for STZ - not hyperglycemic (STZ - not HG) group, and n = 4 mice for STZ - hyperglycemic (STZ - HG) group. Horizontal line indicates mean, and error bars indicate SD. Significance determined with one-way ANOVA and Tukey's multiple comparisons test (B). *p<0.05, **p<0.01.

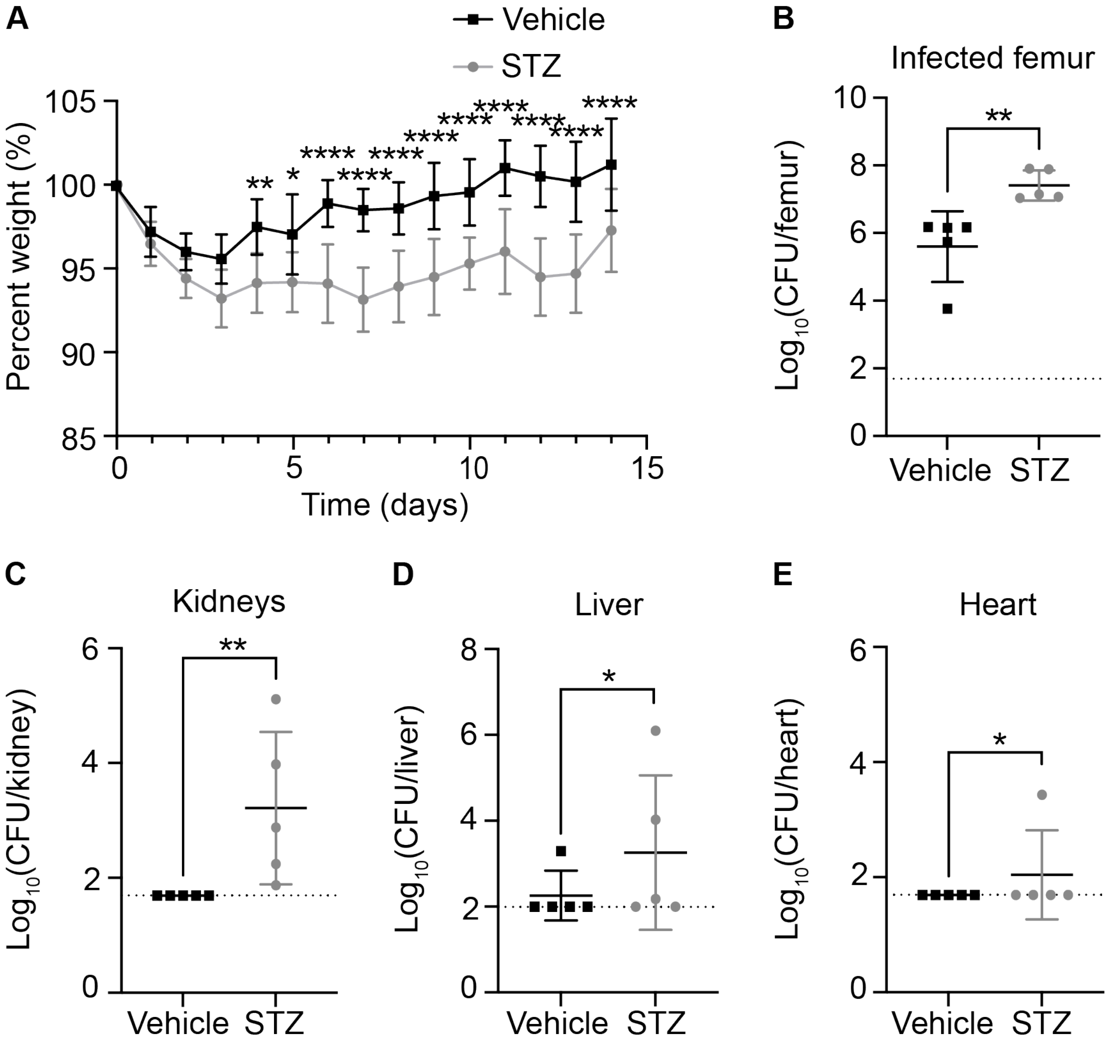

Figure S3. *S. aureus* burdens are increased in mice subjected to chronic hyperglycemia and infected with a lower inoculum. Eight-week old male mice were treated with sodium citrate (vehicle) or streptozotocin (STZ) intraperitoneally for 5 days. 30 days after the final injection, mice were infected with $1x10^5$ CFU of WT *S. aureus* via intraosseous injection. (A) Weights were recorded every 24 hrs and normalized to the starting weight of each animal on the day of infection (percent weight). Mice were sacrificed at day 14 post-infection, and the bacterial burdens (CFU) were enumerated in (B) infected femur, (C) kidneys, (D) liver, and (E) heart. One experiment was conducted with n = 5 mice per group. Dotted lines indicate limit of detection. Horizontal lines indicate mean, and error bars represent SD. Significance

determined with multiple paired t-tests (A) and with Mann-Whitney test (B-E). $^{*}p<0.05$, $^{**}p<0.01$, $^{****}p<0.0001$.

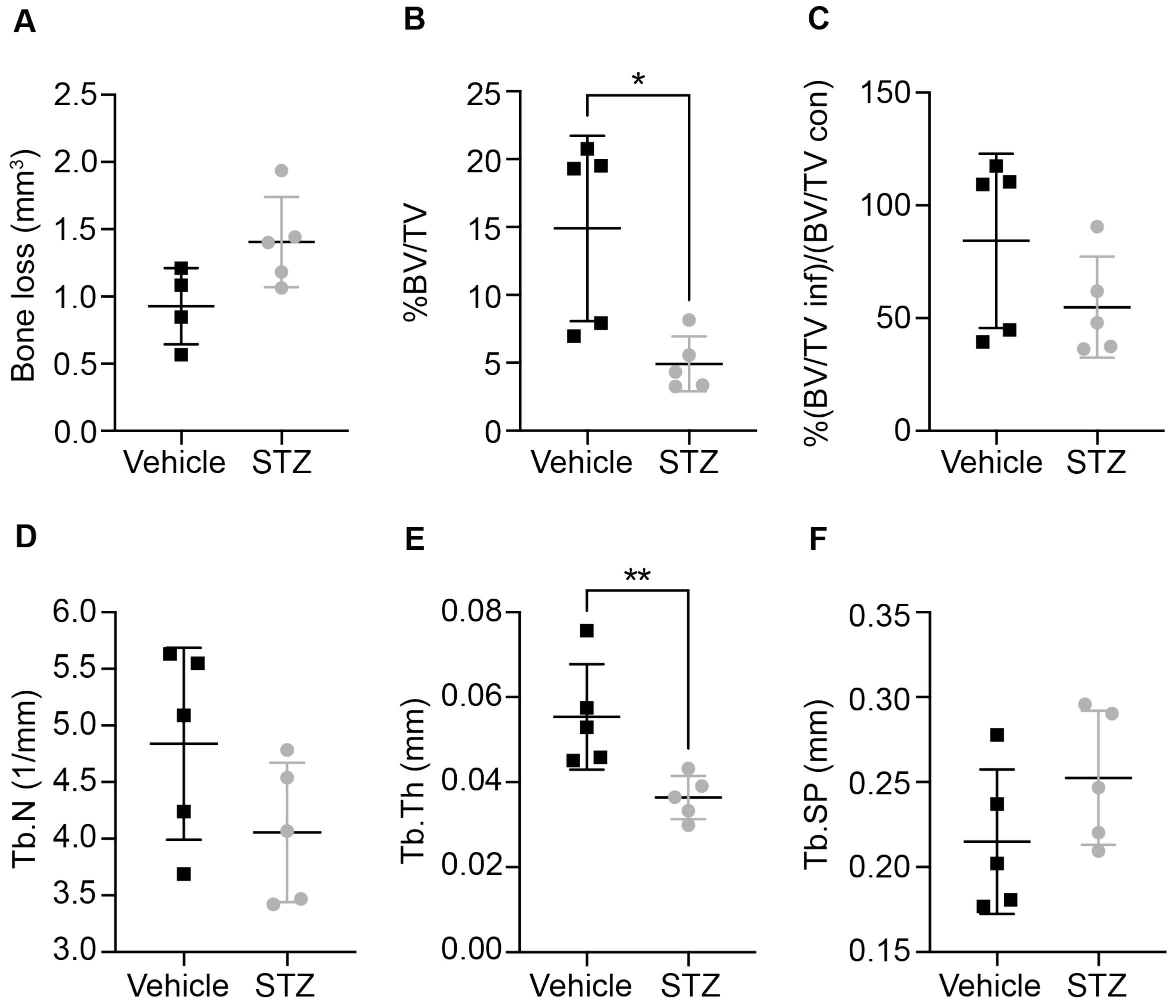

Figure S4. *S. aureus* induces bone destruction in mice subjected to chronic hyperglycemia. Eight-week old male mice were treated with sodium citrate (vehicle) or streptozotocin (STZ) intraperitoneally for 5 days. 30 days after the final injection, mice were infected with $1x10^5$ CFU of WT *S. aureus* via intraosseous injection. At 14 days post-infection, the infected and contralateral femurs were isolated and fixed in neutral buffered formalin prior to micro-computed tomography. (A) Cortical bone loss was calculated with n = 4 vehicle-treated mice and n = 5 STZ-treated mice. (B) Trabecular bone volume divided by total volume (BV/TV) of infected femurs and (C) BV/TV of infected femurs relative to contralateral femurs were quantified. (D) Trabecular number (Tb.N), (E) trabecular thickness (Tb.Th), and (F) trabecular spacing (Tb.Sp) were quantified in infected femurs. One experiment was conducted

and n = 5 mice per group unless otherwise noted. Horizontal lines indicate mean, and error bars represent SD. Significance determined with Mann-Whitney test. *p<0.05, **p<0.01.

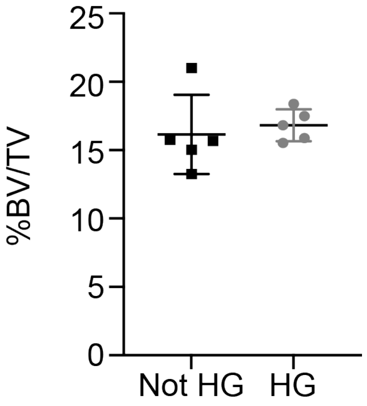

Figure S5. Similar trabecular bone volume is observed in uninfected hyperglycemic and euglycemic mice. Eight-week old male mice were treated with streptozotocin (STZ) intraperitoneally for 5 days. 10 days after the final injection, blood glucose concentration was quantified from a tail vein bleed to identify non-hyperglycemic (Not HG) and hyperglycemic (HG) mice. Left femurs were isolated and fixed in neutral buffered formalin prior to micro-computed tomography. Trabecular bone volume divided by total volume (BV/TV) was quantified. One experiment was conducted with n = 5 mice per group. Horizontal lines indicate mean, and error bars represent SD. Significance determined with Mann-Whitney test.

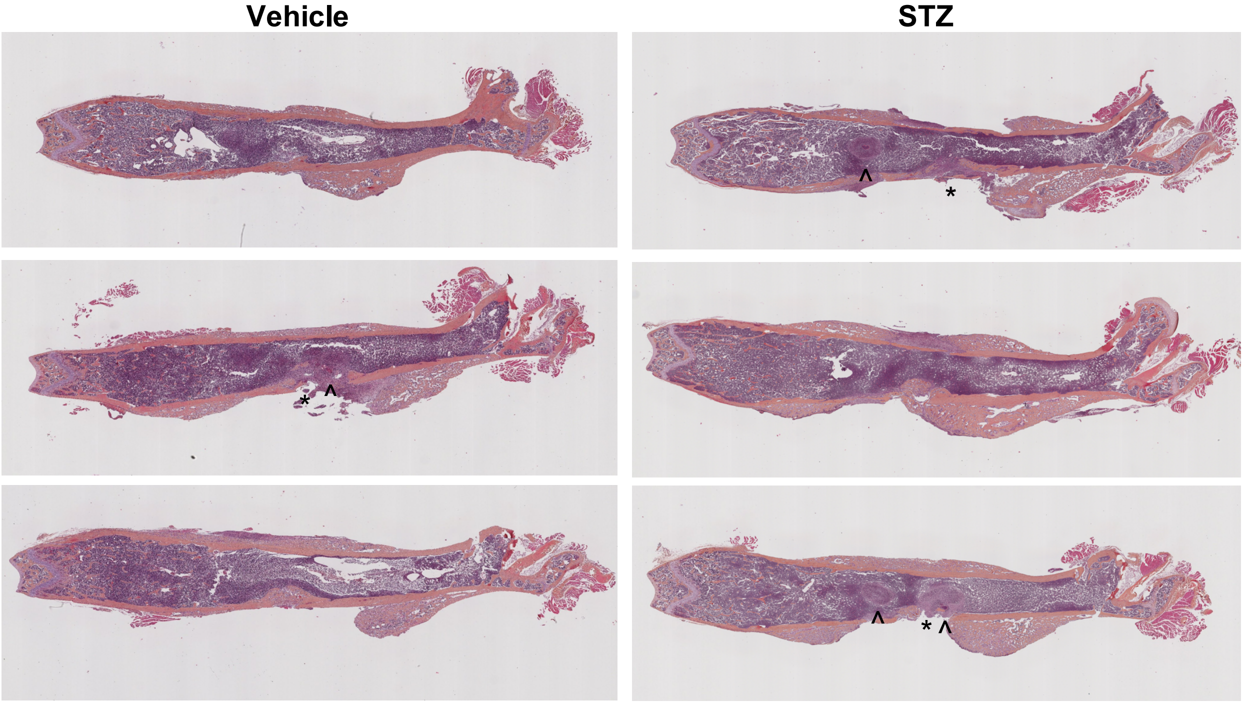

Figure S6. Histological sections of acute hyperglycemic and euglycemic infected femurs. Eight-week old male mice were treated with sodium citrate (vehicle) or streptozotocin (STZ) intraperitoneally for 5 days. 10 days after the final injection, mice were infected with $1x10^5$ CFU of WT *S. aureus* via intraosseous injection. Mice were sacrificed at day 14 post-infection, and the infected femurs were decalcified, sectioned, and stained with H&E. One experiment was conducted with n = 3 mice per group. ^ = *S. aureus* abscess or inflammation suggestive of a staphylococcal abscess community and * = visible cortical bone destruction.

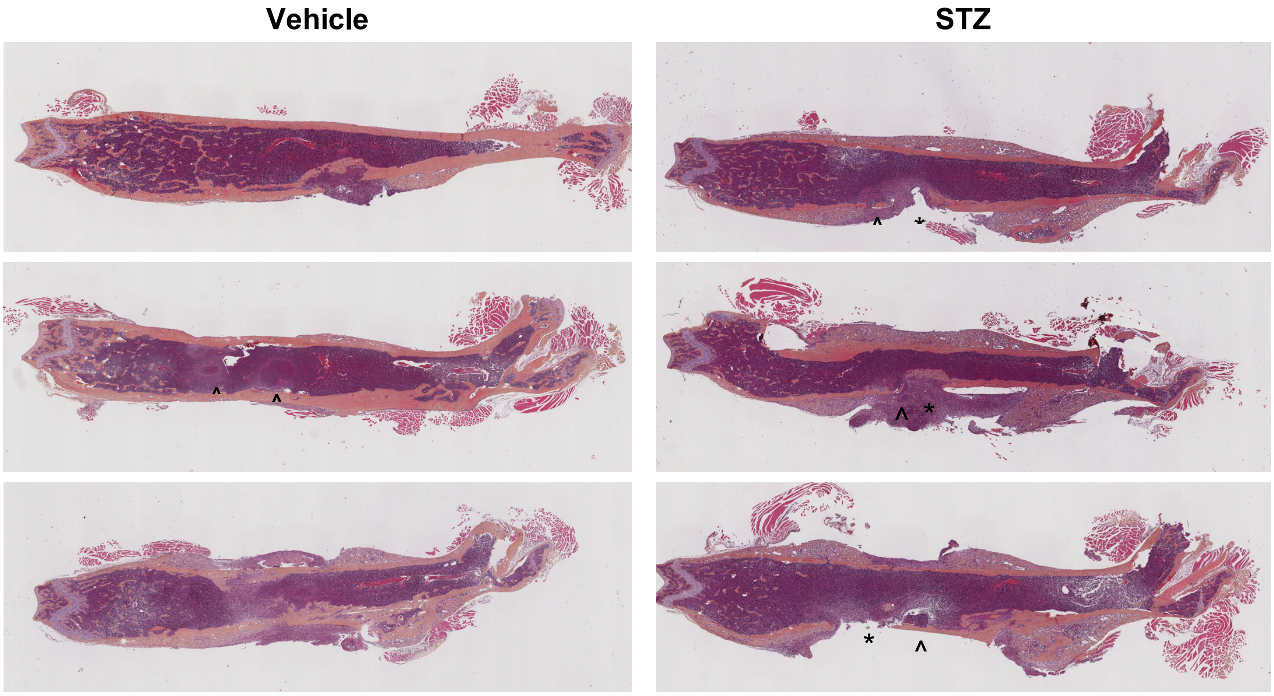

Figure S7. Histological sections of infected femurs from mice with chronic hyperglycemia or euglycemia. Eight-week old male mice were treated with sodium citrate (vehicle) or streptozotocin (STZ) intraperitoneally for 5 days. 30 days after the final injection, mice were infected with $1x10^5$ CFU of WT *S. aureus* via intraosseous injection. Mice were sacrificed at day 14 post-infection, and the infected femurs were decalcified, sectioned, and stained with H&E. One experiment was conducted with n = 3 mice per group. ^ = *S. aureus* abscess or inflammation suggestive of a staphylococcal abscess community and * = visible cortical bone destruction.

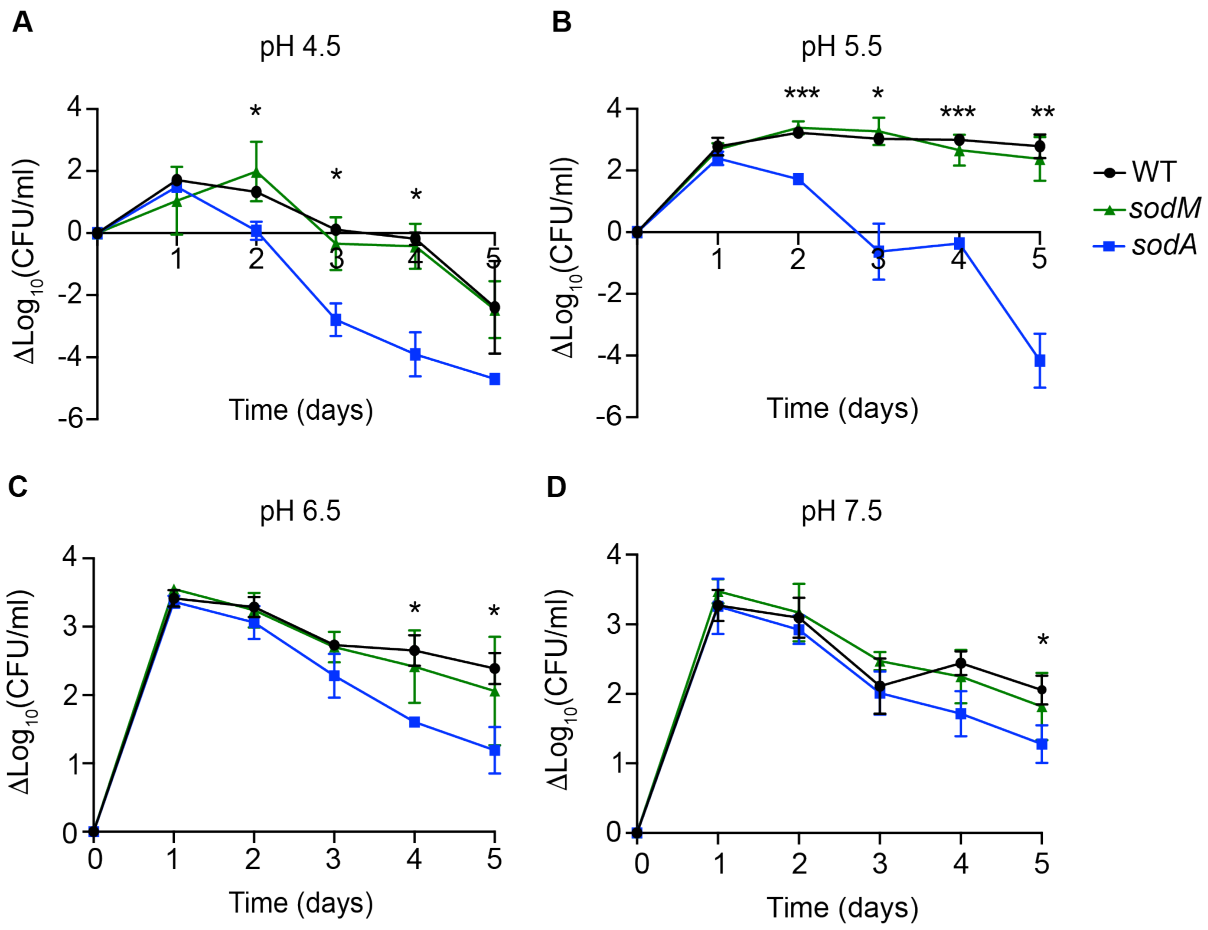

Figure S8. *S. aureus sodA* is required for survival during culture in low pH. WT, *sodA*::tet, and *sodM*::erm *S. aureus* strains were grown in 10 ml TSB at pH (A) 4.5, (B) 5.5, (C) 6.5, and (D) 7.5 in flasks covered with foil (aerobic) and shaking at 37°C. CFU were quantified every 24 hrs over the course of 5 days and normalized to time 0 hr. N = 2 technical replicates, and n = 3 biological replicates. Line represents mean, and error bars represent SD. Significance determined with two-way ANOVA and Dunnett's multiple comparisons test. *p<0.05, **p<0.01, ***p<0.001 *sodA*::tet relative to WT. All comparisons made to WT.

Table S1. *S. aureus* genes identified as essential (Dval<0.01) for bacterial survival during osteomyelitis in hyperglycemic mice by TnSeq analysis. *In vitro* condition n = 2 and vehicle and STZ conditions n = 6. Red indicates Dval<0.01, yellow indicates Dval<0.1.

USA 300 Locus	USA 300 Annotation	Avg *In vitro* Dval	Avg Vehicle Dval	Avg STZ Dval	Dval ratio STZ/Vehicle	Compromised in vitro?	Compromised in Vehicle?
SAUSA300_2214	FmhB protein	0.020991915	0.094326519	0.00025935	0.002749488	X	X
SAUSA300_1887	geranylgeranylglyceryl phosphate synthase family protein, PcrB	0.070869336	0.046393414	0.001800544	0.038810333	X	X
SAUSA300_1682	catabolite control protein A	0.069810323	0.070937935	0.00025935	0.003656007	X	X
SAUSA300_0625	teichoic acid ABC transporter protein	0.029919695	0.031364059	0.00025935	0.008269007	X	X
SAUSA300_0751	tRNA-Arg	1.889152853	1.851989131	0.00025935	0.000140038		
SAUSA300_0348	twin-arginine translocation protein, TatA/E family	0.16848963	0.049664834	0.00025935	0.005221997		X
SAUSA300_1914	GntR family regulatory protein	0.330968221	0.192469964	0.00025935	0.001347481		
SAUSA300_1823	tRNA-Ser	0.060184268	0.024885775	0.002757285	0.110797648	X	X
SAUSA300_1406	phiSLT ORF 104b-like protein	0.74744902	0.068407846	0.003325035	0.048606041		X
SAUSA300_1825	tRNA-Ser	0.048246778	0.028714742	0.003813889	0.132819877	X	X
SAUSA300_1629	threonyl-tRNA synthetase	0.075079895	0.082220633	0.007987498	0.097147126	X	X
SAUSA300_1726	crcB family protein	0.154933519	0.488635818	0.009247294	0.018924716		
SAUSA300_1614	glutamate-1-semialdehyde-2,1-aminomutase	0.085759764	0.260423627	0.00409181	0.015712131	X	
SAUSA300_1513	superoxide dismutase (Mn/Fe family)	0.120544879	0.260160466	0.00025935	0.000996883		
SAUSA300_1593	protein-export membrane protein SecF	0.022864608	0.015902672	0.00045796	0.028797671	X	X
SAUSA300_0763	carboxylesterase	0.010841799	0.023273827	0.005022352	0.215793983	X	X
SAUSA300_1591	adenine phosphoribosyltransferase	0.252611739	0.662257121	0.00025935	0.000391615		
SAUSA300_1095	carbamoyl-phosphate synthase, small subunit	0.037906039	0.094168636	0.00025935	0.002754098	X	X
SAUSA300_0996	dihydrolipoamide dehydrogenase	0.023146317	0.04582584	0.00025935	0.005659462	X	X
SAUSA300_2606	imidazole glycerol phosphate synthase subunit hisF	0.775787559	1.135878683	0.00025935	0.000228325		
SAUSA300_2219	molybdenum cofactor biosynthesis protein A	0.452548966	0.592059977	0.00025935	0.000438046		
SAUSA300_1182	pyruvate ferredoxin oxidoreductase, alpha subunit	0.025617394	0.027474566	0.000492468	0.017924514	X	X
SAUSA300_1195	tRNA delta(2)-isopentenylpyrophosphate transferase	0.085444189	0.075367971	0.000915064	0.012141287	X	X
SAUSA300_2292	isopentenyl-diphosphate delta-isomerase	0.021328702	0.02884698	0.001878126	0.065106502	X	X
SAUSA300_1112	protein phosphatase 2C domain protein	0.254133761	0.083398413	0.002027568	0.024311835		X
SAUSA300_1096	carbamoyl-phosphate synthase, large subunit	0.030947419	0.108403898	0.00258671	0.023861783	X	
SAUSA300_0945	isochorismate synthase family protein	0.089671405	0.094674924	0.008190423	0.086511005	X	X
SAUSA300_0235	L-lactate dehydrogenase	0.150432582	0.074266515	0.009058892	0.12197815		X
SAUSA300_0454	recombination protein RecR	0.095745825	0.558699253	0.00025935	0.000464203	X	
SAUSA300_1039	ribonuclease HIII	0.136490304	0.487391617	0.00025935	0.000532118		
SAUSA300_1468	DNA repair protein RecN	0.034929421	0.082606821	0.001238931	0.014997929	X	X
SAUSA300_1251	DNA topoisomerase IV, subunit A	0.091358919	0.094223246	0.003733489	0.039623862	X	X
SAUSA300_1656	universal stress protein family	0.05378754	0.366091114	0.007202291	0.019673494	X	
SAUSA300_0006	DNA gyrase, A subunit	0.029066593	0.024980373	0.007379942	0.29542963	X	X
SAUSA300_1420	conserved hypothetical phage protein	0.4880503	0.128728995	0.00025935	0.002014695		
SAUSA300_0792	conserved hypothetical protein	1.541986809	6.170506545	0.00025935	4.20305E-05		
SAUSA300_0933	conserved hypothetical protein	0.025345578	5.464665996	0.00025935	4.74594E-05	X	
SAUSA300_1794	conserved hypothetical protein	0.087631903	3.072085113	0.00025935	8.44214E-05	X	
SAUSA300_1552	conserved hypothetical protein	0.156728111	1.541411799	0.00025935	0.000168255		
SAUSA300_0931	conserved hypothetical protein	2.127720434	1.236444995	0.00025935	0.000209754		
SAUSA300_1797	conserved hypothetical protein	0.515071025	0.989799861	0.00025935	0.000262022		
SAUSA300_0957	conserved hypothetical protein	0.108430987	0.974442154	0.00025935	0.000266152		
SAUSA300_1175	conserved hypothetical protein	0.034303278	0.831389257	0.00025935	0.000311947	X	
SAUSA300_1012	conserved hypothetical protein	0.507879292	0.744755314	0.00025935	0.000348235		
SAUSA300_1040	conserved hypothetical protein	0.468988469	0.575949279	0.00025935	0.000450299		
SAUSA300_1721	conserved hypothetical protein	0.357157659	0.445423396	0.00025935	0.000582254		
SAUSA300_1215	conserved hypothetical protein	1.421378703	0.345528858	0.00025935	0.000750587		
SAUSA300_0937	conserved hypothetical protein	0.093906093	0.317384979	0.00025935	0.000817145	X	
SAUSA300_1572	conserved hypothetical protein	0.082547995	0.302665078	0.00025935	0.000856887	X	
SAUSA300_0847	conserved hypothetical protein	0.029609372	0.262979047	0.00025935	0.000986199	X	
SAUSA300_0997	conserved hypothetical protein	0.216547238	0.21016794	0.00025935	0.001234011		
SAUSA300_1010	conserved hypothetical protein	0.764604237	0.204717532	0.00025935	0.001266866		
SAUSA300_1084	conserved hypothetical protein	1.26286161	0.161939258	0.00025935	0.001601524		
SAUSA300_2132	conserved hypothetical protein	0.148202578	0.117905356	0.00025935	0.002199642		
SAUSA300_1204	conserved hypothetical protein	0.422677465	0.111673209	0.00025935	0.002322398		
SAUSA300_0906	conserved hypothetical protein	0.070339456	0.033978822	0.00025935	0.007632684	X	X
SAUSA300_2547	conserved hypothetical protein	0.095537371	0.028611597	0.00025935	0.009064493	X	X
SAUSA300_0857	conserved hypothetical protein	0.091275779	0.02129396	0.00025935	0.012179492	X	X
SAUSA300_1935	phi77 ORF029-like protein	0.109362926	0.691880947	0.00025935	0.000374847		
SAUSA300_1429	phiSLT ORF53-like protein	0.397280812	0.737758677	0.00025935	0.000351537		
SAUSA300_1411	phiSLT ORF66-like protein	0.357076945	1.630466604	0.00025935	0.000159065		
SAUSA300_0663	putative lipoprotein	0.127588782	0.542002141	0.00025935	0.000478503		
SAUSA300_1492	putative lipoprotein	0.034035853	0.016867858	0.00025935	0.015375374	X	X
SAUSA300_0035	truncated hypothetical protein	0.660124585	1.710639102	0.00025935	0.00015161		
SAUSA300_2143	conserved hypothetical protein	0.505895254	0.887672785	0.000579076	0.000652353		
SAUSA300_1414	phiSLT ORF 78B-like protein	0.241319969	0.228570322	0.001695388	0.007417358		
SAUSA300_1757	serine protease SplB	0.611947673	0.29361746	0.002147651	0.007314453		
SAUSA300_0026	conserved hypothetical protein OrfX	0.180468185	0.532053966	0.005190957	0.009756448		
SAUSA300_1924	holin	1.013877153	0.961253799	0.006689285	0.006958916		
SAUSA300_0920	conserved hypothetical protein	0.88238289	0.264643647	0.008888675	0.033587336		
SAUSA300_1342	conserved hypothetical protein	0.331375464	0.711509767	0.009564624	0.013442717		

Table S2. *S. aureus* transposon mutants with compromised fitness (Dval>0.01 and <0.1) during osteomyelitis in hyperglycemic mice but not *in vitro* or in euglycemic infection, as identified by TnSeq analysis. *In vitro* condition n = 2 and vehicle and STZ conditions n = 6. Yellow indicates Dval<0.1.

USA 300 Locus	USA 300 Annotation	Avg *In vitro* Dval	Avg Vehicle Dval	Avg STZ Dval	Dval ratio STZ/Vehicle
SAUSA300_0308	ABC transporter, permease protein	0.490129267	0.616006153	0.04638433	0.075298485
SAUSA300_1345	asparaginyl-tRNA synthetase	0.28993173	0.384617317	0.089353624	0.232318256
SAUSA300_0513	glutamyl-tRNA synthetase	0.228018682	0.345086043	0.07992978	0.231622754
SAUSA300_2033	K+-transporting ATPase, B subunit	0.659266028	0.858809426	0.095885776	0.111649655
SAUSA300_0988	potassium uptake protein	0.237775911	0.111706618	0.057297718	0.512930385
SAUSA300_1170	transcriptional regulator, GntR family	0.142635526	0.165520727	0.025030655	0.151223692
SAUSA300_0447	tRNA-Ser	0.20266131	0.159350456	0.017772006	0.1115278
SAUSA300_1866	two-component sensor histidine kinase	0.332279995	0.136079582	0.052853823	0.388403769
SAUSA300_0577	putative transcriptional regulator	0.420486787	0.335787199	0.057317191	0.170694986
SAUSA300_2563	putative transcriptional regulator	0.383700014	0.527249454	0.061897954	0.117397853
SAUSA300_0195	RpiR family transcriptional regulator	0.54536562	0.546072748	0.063949659	0.117108314
SAUSA300_1455	transcriptional regulator, AraC family	0.196767634	0.658233132	0.011568928	0.01757573
SAUSA300_1007	inositol monophosphatase family protein	0.593825633	0.462676534	0.048941843	0.105779824
SAUSA300_1512	penicillin-binding protein 3	0.171050585	0.215631267	0.084985941	0.394126242
SAUSA300_1055	fibrinogen-binding protein	0.179843532	0.394810617	0.08113842	0.205512254
SAUSA300_0116	iron compound ABC transporter, permease protein SirB	0.64608191	0.345858849	0.088075879	0.254658452
SAUSA300_0344	putative lipoprotein	0.872103781	0.198330697	0.012148062	0.061251548
SAUSA300_0999	spermidine/putrescine ABC transporter, ATP-binding protein	0.141300437	0.241180257	0.071312061	0.295679514
SAUSA300_1001	spermidine/putrescine ABC transporter, permease protein	0.29457968	0.563537419	0.068006349	0.12067761
SAUSA300_1002	spermidine/putrescine ABC transporter, spermidine/putrescine-binding protein	0.470846857	0.116560701	0.078960879	0.677422818
SAUSA300_1299	putative tellurite resistance protein	0.54102266	0.419832776	0.034791561	0.082870046
SAUSA300_2485	methylated DNA-protein cysteine methyltransferase	0.176764323	0.791211693	0.062870275	0.07946075
SAUSA300_1605	rod shape-determining protein MreC	0.202646722	0.28830262	0.043707016	0.151601175
SAUSA300_1305	2-oxoglutarate dehydrogenase, E2 component, dihydrolipoamide succinyltransferase	0.679241823	0.319254525	0.07389339	0.231456045
SAUSA300_1564	acetyl-CoA carboxylase, biotin carboxyl carrier protein	0.667675803	0.543935123	0.069754138	0.128239812
SAUSA300_1313	carboxyl-terminal protease	0.242227458	1.072594694	0.036930222	0.034430733
SAUSA300_0838	D-alanine-activating enzyme/D-alanine-D-alanyl, dltD protein	0.17974059	0.251368359	0.0793988	0.315866325
SAUSA300_0697	exsB protein	0.112322392	0.142136076	0.040576882	0.285479118
SAUSA300_2225	molybdenum cofactor biosynthesis protein C	0.375367234	0.965802373	0.056287487	0.058280544
SAUSA300_0825	oxidoreductase, 2-nitropropane dioxygenase family	0.659249281	0.159186387	0.054550817	0.342685185
SAUSA300_0970	phosphoribosylformylglycinamidine synthase I	0.376887983	1.460463933	0.08585366	0.058785197
SAUSA300_1491	proline dipeptidase	0.354546449	0.266255534	0.057604766	0.216351432
SAUSA300_1315	PTS system, glucose-specific IIA component	0.249049869	1.066831826	0.010480373	0.009823829
SAUSA300_1139	succinyl-CoA synthetase, alpha subunit	0.262959204	5.560403062	0.042623393	0.007665522
SAUSA300_0665	acetyltransferase, GNAT family	1.520691788	1.170260557	0.015634564	0.0133599
SAUSA300_0441	acetyltransferase, GNAT family	0.482284534	0.120050847	0.099452841	0.828422646
SAUSA300_1428	conserved hypothetical phage protein	0.48799898	0.101006121	0.068385681	0.677044911
SAUSA300_1853	conserved hypothetical protein	0.942138437	0.851636431	0.010672903	0.01253223
SAUSA300_1325	conserved hypothetical protein	0.134563734	0.122955109	0.012574423	0.102268404
SAUSA300_2529	conserved hypothetical protein	0.612560461	0.694862082	0.013381314	0.019257511
SAUSA300_0465	conserved hypothetical protein	0.604635956	1.972033824	0.016821858	0.008530208
SAUSA300_0243	conserved hypothetical protein	0.969005409	0.181318046	0.028888277	0.159323782
SAUSA300_1053	conserved hypothetical protein	0.197818757	0.148849753	0.0366234	0.246042731
SAUSA300_1692	conserved hypothetical protein	0.275412435	1.359637166	0.037299294	0.02743327
SAUSA300_0463	conserved hypothetical protein	0.270556045	1.057420462	0.039995047	0.037823221
SAUSA300_1203	conserved hypothetical protein	0.531534288	0.776550662	0.048808058	0.062852381
SAUSA300_1706	conserved hypothetical protein	0.444288959	0.406692208	0.068359788	0.168087283
SAUSA300_1041	conserved hypothetical protein	0.18760876	0.817839638	0.076825237	0.093936798
SAUSA300_0655	conserved hypothetical protein	0.531852992	0.280626734	0.078598402	0.280081661
SAUSA300_1100	conserved hypothetical protein	0.581057373	0.301069795	0.079501488	0.264063314
SAUSA300_1277	conserved hypothetical protein	0.881983734	0.32147742	0.091989402	0.286145763
SAUSA300_0831	conserved hypothetical protein	0.294392218	0.31983834	0.092847751	0.290295877
SAUSA300_0011	conserved hypothetical protein	0.378838704	0.271835843	0.093822136	0.345142622
SAUSA300_1937	phi77 ORF045-like protein	1.694587616	1.207502983	0.043863462	0.036325759
SAUSA300_1405	phiSLT ORF 101-like protein, terminase, small subunit	0.526834837	0.15704799	0.092398826	0.588347714
SAUSA300_1399	phiSLT ORF110-like protein	0.763083833	0.101516588	0.055925305	0.550898196
SAUSA300_1395	phiSLT ORF116b-like protein	0.584689284	1.364661215	0.08633539	0.063265072
SAUSA300_1376	putative lipoprotein	0.662293021	0.510554721	0.014378699	0.028162896
SAUSA300_0913	putative membrane protein	0.449496615	0.540341288	0.081939014	0.151643074
SAUSA300_0316	ROK family protein	0.817666261	0.589156867	0.069467005	0.117909183
SAUSA300_0658	transcriptional regulator, LysR family	0.160756131	0.430252834	0.082636901	0.192065907
